# Epigenetic priming enhances antitumor immunity in platinum-resistant ovarian cancer

**DOI:** 10.1172/JCI158800

**Published:** 2022-07-15

**Authors:** Siqi Chen, Ping Xie, Matthew Cowan, Hao Huang, Horacio Cardenas, Russell Keathley, Edward J. Tanner, Gini F. Fleming, John W. Moroney, Alok Pant, Azza M. Akasha, Ramana V. Davuluri, Masha Kocherginsky, Bin Zhang, Daniela Matei

**Affiliations:** 1 Department of Medicine, Hematology/Oncology Division,; 2 Department of Obstetrics and Gynecology,; 3 Driskill Graduate Training Program in Life Sciences, and; 4 Robert H. Lurie Comprehensive Cancer Center, Feinberg School of Medicine, Northwestern University, Chicago, Illinois, USA.; 5 Department of Medicine, Hematology/Oncology Division, University of Chicago, Chicago, Illinois, USA.; 6 Northwestern Medicine, Lake Forest Hospital, Lake Forest, Illinois, USA.; 7 Department of Biomedical Informatics, Stony Brook University, Stony Brook, New York, USA.; 8 Division of Biostatistics, Department of Preventive Medicine, Feinberg School of Medicine, Northwestern University, Chicago, Illinois, USA.; 9 Jesse Brown VA Medical Center, Chicago, Illinois, USA.

**Keywords:** Immunology, Oncology, Cancer, Cancer immunotherapy, Epigenetics

## Abstract

**Background:**

Immune checkpoint inhibitors (ICIs) have modest activity in ovarian cancer (OC). To augment their activity, we used priming with the hypomethylating agent guadecitabine in a phase II study.

**Methods:**

Eligible patients had platinum-resistant OC, normal organ function, measurable disease, and received up to 5 prior regimens. The treatment included guadecitabine (30 mg/m^2^) on days 1–4, and pembrolizumab (200 mg i.v.) on day 5, every 21 days. The primary endpoint was the response rate. Tumor biopsies, plasma, and PBMCs were obtained at baseline and after treatment.

**Results:**

Among 35 evaluable patients, 3 patients had partial responses (8.6%), and 8 (22.9%) patients had stable disease, resulting in a clinical benefit rate of 31.4% (95% CI: 16.9%–49.3%). The median duration of clinical benefit was 6.8 months. Long-interspersed element 1 (LINE1) was hypomethylated in post-treatment PBMCs, and methylomic and transcriptomic analyses showed activation of antitumor immunity in post-treatment biopsies. High-dimensional immune profiling of PBMCs showed a higher frequency of naive and/or central memory CD4^+^ T cells and of classical monocytes in patients with a durable clinical benefit or response (CBR). A higher baseline density of CD8^+^ T cells and CD20^+^ B cells and the presence of tertiary lymphoid structures in tumors were associated with a durable CBR.

**Conclusion:**

Epigenetic priming using a hypomethylating agent with an ICI was feasible and resulted in a durable clinical benefit associated with immune responses in selected patients with recurrent OC.

**Trial registration:**

ClinicalTrials.gov NCT02901899.

**Funding:**

US Army Medical Research and Material Command/Congressionally Directed Medical Research Programs (USAMRMC/CDMRP) grant W81XWH-17-0141; the Diana Princess of Wales Endowed Professorship and LCCTRAC funds from the Robert H. Lurie Comprehensive Cancer Center; Walter S. and Lucienne Driskill Immunotherapy Research funds; Astex Pharmaceuticals; Merck & Co.; National Cancer Institute (NCI), NIH grants CCSG P30 CA060553, CCSG P30 CA060553, and CA060553.

## Graphical abstract

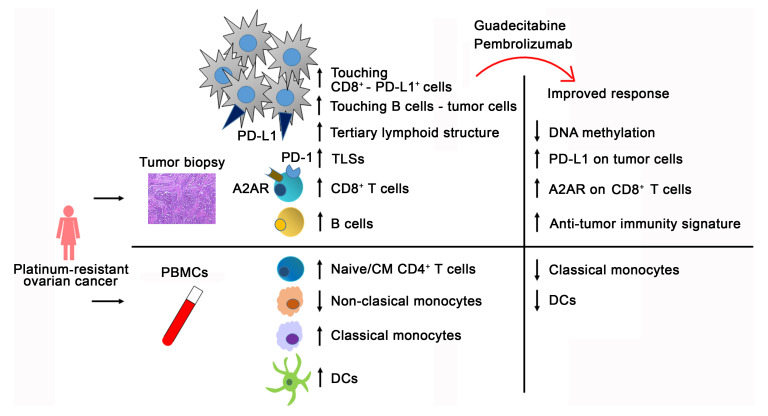

## Introduction

Strategies targeting immune checkpoints have significantly altered the clinical outcomes of various malignancies and completely altered the standard-of-care approach (1). Ovarian cancer (OC) stands out as one of the few tumor types for which immunotherapy has failed to make a positive impact (2–5). It has been postulated that potent immunosuppressive signals dominate the tumor microenvironment (TME) of ovarian tumors. Key inducers of the “cold” milieu of OC remain controversial, and interventions to overcome the antitumor immunity barrier are lagging. Epigenetic modifications, specifically methylation of CpG islands, have been shown to silence tumor antigens (TAs) (6), repress highly immunogenic endogenous retroviral genes (7, 8), and downregulate programmed death ligands (PD-L1 and PD-L2) (9). Collectively, these findings have fueled the hypothesis that targeting epigenetic mechanisms could increase the immunogenicity of OC and augment the activity of immune checkpoint inhibitors (ICIs) (10, 11). The goals of the current study were to develop a novel combination regimen that enhances the activity of an ICI through inhibition of DNA methylation and to identify markers of immune activation in vivo.

Hypomethylating agents (HMAs) like decitabine and 5-azacitadine have been shown to restore the expression of epigenetically silenced genes, including tumor suppressor genes (12) and TAs (13) in a variety of cancers, including OC. The cancer-testis antigen NY-ESO-1, which is epigenetically silenced in ovarian tumors, was reexpressed in response to a HMA (13, 14). In a phase I study, the HMA decitabine potentiated the effects of a NY-ESO-1 vaccine, augmenting T cell immune responses and inducing antitumor activity (15). HMAs were also shown to induce immune signaling in cancer cells by augmenting the viral response pathway and inducing reexpression of endogenous retroviral genes incorporated into the human genome (7, 8). Epigenetically repressed Th cell responses could be restored by epigenetic modulators in immunocompetent OC mouse models, in which the response to ICIs was also augmented by this strategy (16). In previous clinical trials for patients with OC using decitabine or guadecitabine as resensitizers to carboplatin, we observed a strong transcriptomic immune signature emerging in vivo after treatment with HMAs (17, 18). Together these preclinical and early clinical results supported the hypothesis that interventions targeting the epigenome could elicit an inflamed tumor milieu and provided a strong rationale for this study.

Here, we describe the clinical and biological activity of guadecitabine, a second-generation HMA, given at a low dose as a priming strategy before pembrolizumab, a humanized anti–programmed cell death 1 (anti–PD-1) antibody in a clinical trial for patients with recurrent, platinum-resistant OC. The methylomic and transcriptomic effects of the combination demonstrated activation of antitumor immunity. High-dimensional immune profiling of PBMCs and of tumor biopsies obtained before and after treatment showed distinct immune profiles and tissue architectural features associated with clinical benefit. Our study provides an in-depth view of the immune milieu of platinum-resistant OC and of the effects of the combination of HMAs and pembrolizumab on the interactions between immune cell populations and tumor cells.

## Results

### Clinical

**Figure 1:**
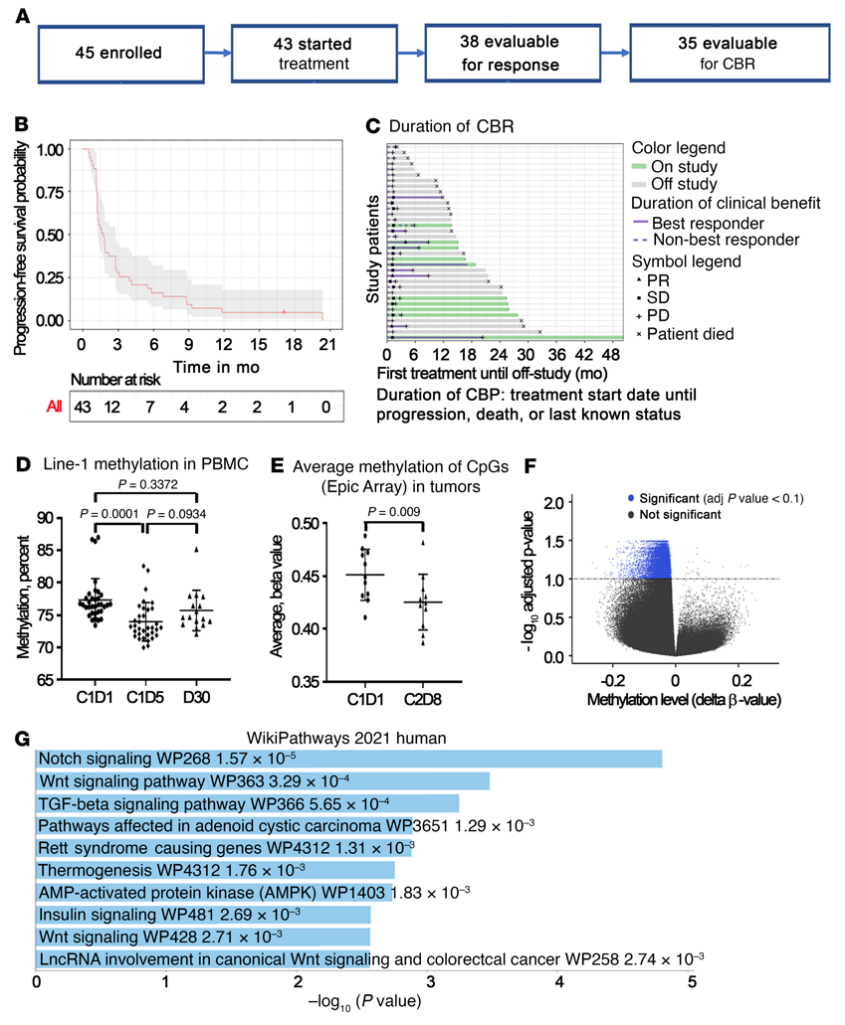
G+P in recurrent OC. (**A**) CONSORT diagram. CBR, clinical benefit or response. (**B**) Kaplan-Meier estimates of PFS (*n =* 43). (**C**) Duration of clinical benefit or response (months). PD, progressive disease. (**D**) LINE-1 methylation in PBMCs before G+P treatment (C1D1), after G+P treatment (C1D5), and 30 days after treatment discontinuation (D30). Data indicate the mean ± standard deviation. *P* values were determined with a mixed-effects model and Tukey’s multiple-comparison test. *n =* 34 pairs for C1D1 versus C1D5; *n =* 16 pairs for C1D1 versus D30. (**E**) Average methylation of CpGs (β values) measured using Epic arrays in C2D8 versus C1D1 tumor biopsies. Data indicate the mean ± standard deviation. *P* value was determined by paired *t* test. *n =* 11 pairs. (**F**) Volcano plot of DNA methylation (β values) in C2D8 versus C1D1 tumor biopsies (*n =* 11 pairs). adj, adjusted. (**G**) Top 10 pathways identified by WikiPathways enrichment analysis using 767 genes containing DM CpGs associated with CpG islands located in the gene body or promoter-associated region TSS200+TSS1500 (Illumina nomenclature) in C2D8 versus C1D1 tumor biopsies (*n =* 11 pairs).

Between November 9, 2016 and November 25, 2019, 45 patients were enrolled, 43 received at least 1 cycle of treatment, 38 were evaluable for a response on the basis of Response Evaluation Criteria in Solid Tumors (RECIST), version 1.1, and 35 received more than 2 cycles of treatment and were evaluable for a clinical benefit or response (CBR) (Figure 1A). The treatment schema is shown in Supplemental Figure 1A. For the 43 patients who started treatment, the median age was 63.0 years (range, 40–88 years), and most patients were non-Hispanic White individuals (Supplemental Table 1). The median number of prior therapies was 5 (range, 1–11), and the median number of prior platinum-based therapies was 2 (range, 1–4; Supplemental Table 1). The overall response rate was 7.9% (3 of 38); 3 patients had a partial response (PR) (7.9%), and no complete responses (CRs) were recorded (Supplemental Figure 1B). At 3 months, there were 3 sustained PRs, and an additional 8 patients were found to have stable disease (SD), resulting in a clinical benefit rate of 31.4% (95% CI: 16.9%–49.3%). Nine patients were categorized as having a durable clinical benefit or response. The median duration of the clinical benefit was 6.8 months (95% CI: 4.14–11.8 and Figure 1C and Supplemental Figure 1C). Progression-free survival (PFS) at 6 months was 16.3% (95% CI: 8.3%–32.1%), and the median PFS was 1.74 months (95% CI: 1.25–2.76 months, Figure 1B). OS at 24 months was 37.7% (95% CI: 23.3%–61.0%), with a median OS of 16.3 months (95% CI: 11.8 – 28.6 months; Supplemental Figure 1D).

#### Toxicity.

We evaluated toxicity in the 43 patients who received at least 1 cycle of treatment. A total of 41 (95.3%) patients had adverse events (AEs) related to one of the study drugs; 40 (93.0%) patients experienced guadecitabine-related AEs; and 32 (74.4%) patients experienced pembrolizumab-related AEs (Supplemental Table 2). Grade 3/4 AEs were observed in 24 (55.8%) patients and were more commonly attributed to guadecitabine (*n =* 21, 48.8%) rather than to pembrolizumab (*n =* 8, 18.6%). Conversely, 6 patients (14.4%) experienced 6 serious AEs (SAEs) attributed to pembrolizumab, whereas 3 patients (7%) experienced 5 SAEs attributed to guadecitabine. Grade 3/4 SAEs attributed to guadecitabine occurred in 3 (7.0%) patients and included neutropenia, febrile neutropenia, otitis media, and skin infection. Five patients (11.6%) had grade 3/4 SAEs attributed to pembrolizumab, including neutropenia, febrile neutropenia, colitis, arthritis, and a thromboembolic event (Supplemental Table 3).

#### Methylome changes.

The effects of the treatment regimen on DNA methylation were assessed by measuring long-interspersed element 1 (LINE-1) methylation, a reliable indicator of genome-wide methylation (19, 20) in PBMCs using pyrosequencing. A 3.4% overall decrease on cycle 1, day 5 (C1D5) compared with pretreatment (C1D1, *n =* 34 pairs) LINE-1 levels was significant (*P <* 0.001) and remained decreased compared with baseline (*P* = 0.11, *n =* 16 pairs) 30 days after the end of therapy (Figure 1D). We used Infinium EPIC arrays to assess the methylation of more than 800,000 CpGs in 11 paired tumors (C2D8 vs. C1D1). An analysis of the average methylation levels (β values) of all CpGs per sample indicated an overall decrease of approximately 2.6% (*P =* 0.009) in tumors collected after versus before treatment (Figure 1E). An analysis of methylation β values showed that 11,407 CpGs were differentially methylated (DM) in C2D8 versus C1D1 tumors and that most differences (11,382 events) were due to demethylation as depicted in the volcano plot in Figure 1F. The majority of DM CpGs were mapped to the open sea (64.5%) or shore (15.1%, Supplemental Figure 1E). Distribution of DM CpGs by gene region indicated that 64.1% were in the gene body. A number of genes (*n* = 767) had DM CpGs associated with CpG islands within the gene body or the promoter-associated region comprising 1500 bp upstream of the transcription start site (TSS200 plus TSS1500). Genes with the highest numbers of DM CpG sites in promoter-associated regions are listed in Supplemental Figure 1F, including *SLC25A31* (apoptosis), *ASB2* (Notch signaling, T cell development), *DOCK6* (Wnt signaling, metastasis), *NCK2* (proliferation), and others. Pathway enrichment analysis based on the 767 genes containing DM CpGs associated with the gene body or promoter-associated regions showed Notch, Wnt, and TGF-β signaling as the top 3 most significantly enriched pathways (Figure 1G).

#### Effects on the transcriptome.

**Figure 2:**
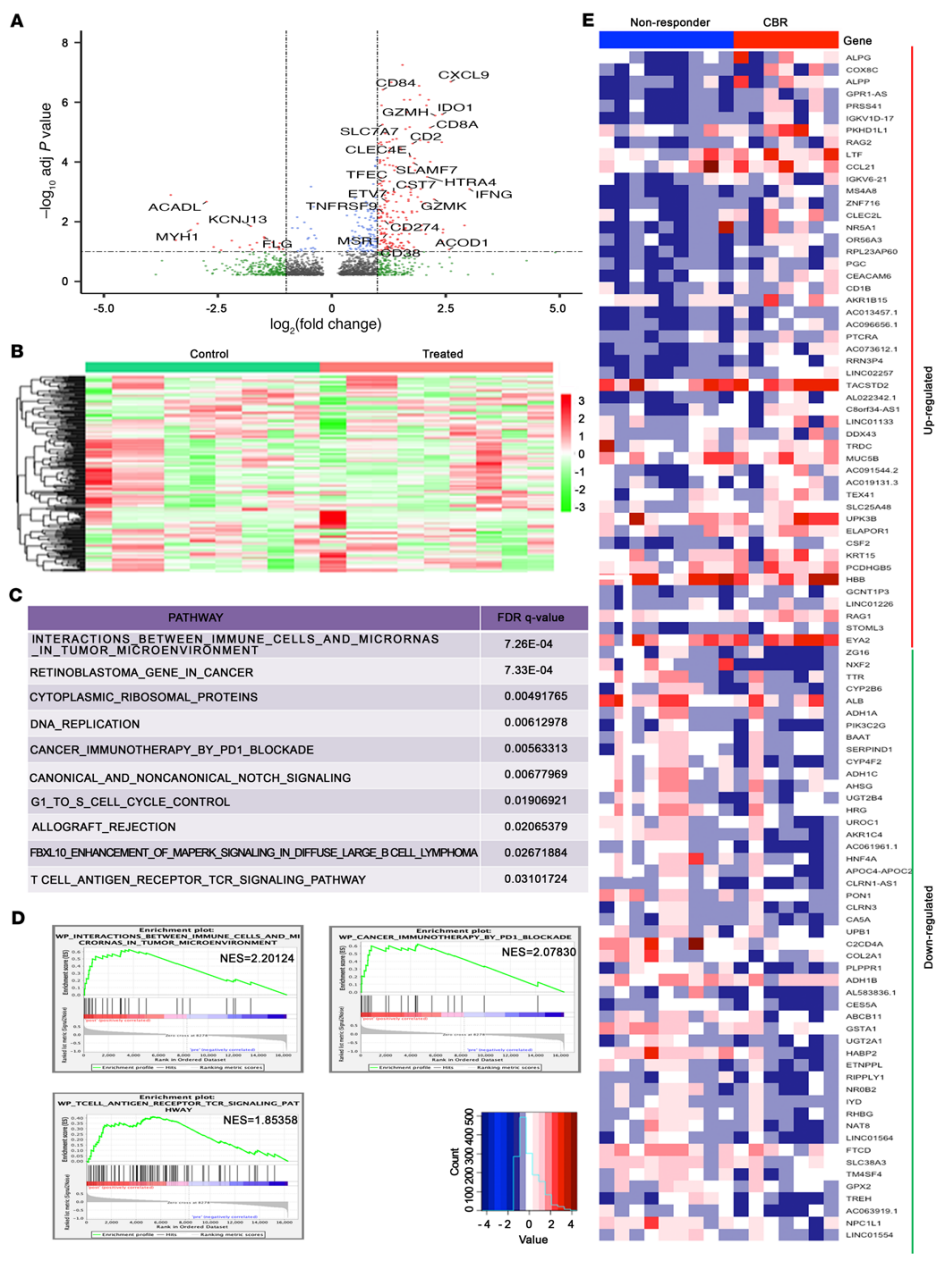
Transcriptomic changes induced by G+P treatment in recurrent OC. (**A**) Volcano plot shows DEGs in post-treatment (C2D8) versus pretreatment (C1D1) tumor biopsies (*n =* 9 pairs). (**B**) Hierarchical clustering and heatmap show the 300 most variable genes between pre- and post-treatment biopsies. (**C**) Top 10 enriched biological pathways determined by GSEA of DEGs between pre- and post-treatment biopsies. (**D**) GSEA enrichment plots for the following biological pathways: “interactions between immune cells and microRNAs in tumor microenvironment,” “cancer immunotherapy by the PD1 blockade,” and “T cell antigen receptor TCR signaling pathway.” NES, normalized enrichment score. (**E**) Heatmap showing mRNA expression levels of the top 50 up- and downregulated genes (FDR <0.10) in pretreatment tumors from patients categorized as responders (durable CBR, *n =* 9) versus nonresponders (*n =* 7).

To assess whether changes in DNA methylation induced by guadecitabine significantly altered transcription, we performed RNA-Seq to compare gene expression in 9 paired tumor biopsies. We found that 330 genes were differentially expressed (FDR <0.10) in C2D8 versus C1D1 tumors, represented as red dots in Figure 2A (volcano plot) and depicted by hierarchical clustering in Figure 2B. Notably, the majority (*n* = 289) of differentially expressed genes (DEGs) were upregulated (Figure 2A), with the top upregulated and downregulated genes listed in Supplemental Tables 4 and 5. The most upregulated genes were *IFNG* (8.1-fold), *PNMA5* (7.5-fold), *CXCL9* (6.1-fold), *CXCR6,* *IL21*, and granzyme H, K, A, and B (Supplemental Table 4), all of which are related to an activated immune response. Interestingly, some transcript, such as *IDO1* and *LAG3*, that negatively affect antitumor immunity were also upregulated in post-treatment specimens, reflecting either compensatory mechanisms or nonspecific global effects of the HMA. Gene set enrichment analysis (GSEA) revealed pathways involved in the immune system, retinoblastoma in cancer, DNA replication, the cell cycle, and other processes as among the top enriched pathways. Specifically, enrichment of the immune system pathways “cancer immunotherapy by PD1 blockade,” “T cell antigen receptor TCR signaling pathway,” and “interactions between immune cells and microRNAs in tumor microenvironment” reflect the activity of this regimen (Figure 2, C and D). We observed distinct transcriptomic profiles in baseline tumors harvested from patients with a durable CBR (*n =* 7) versus those from nonresponders (*n =* 9, Figure 2E). A total of 88 transcripts were significantly upregulated and 188 were downregulated at baseline in tumors from patients in the durable CBR category compared with those from nonresponders (Supplemental Figure 2, A and B, and Supplemental Tables 6 and 7). It has been reported that HMAs could cause the reexpression of endogenous retroviruses, which in turn trigger an IFN response (7). The transcriptomes of 9 paired specimens obtained before and after treatment were analyzed for differential expression of transposable elements (TEs) (see Supplemental Methods) (21, 22). We found that 21 TEs were differentially expressed between pre- and post-treatment specimens (FDR <0.05; Supplemental Table 8), supporting the idea that changes in the expression of retroviral elements are detectable in human tissue after HMA treatment.

#### Immune profiles associated with a response.

**Figure 3:**
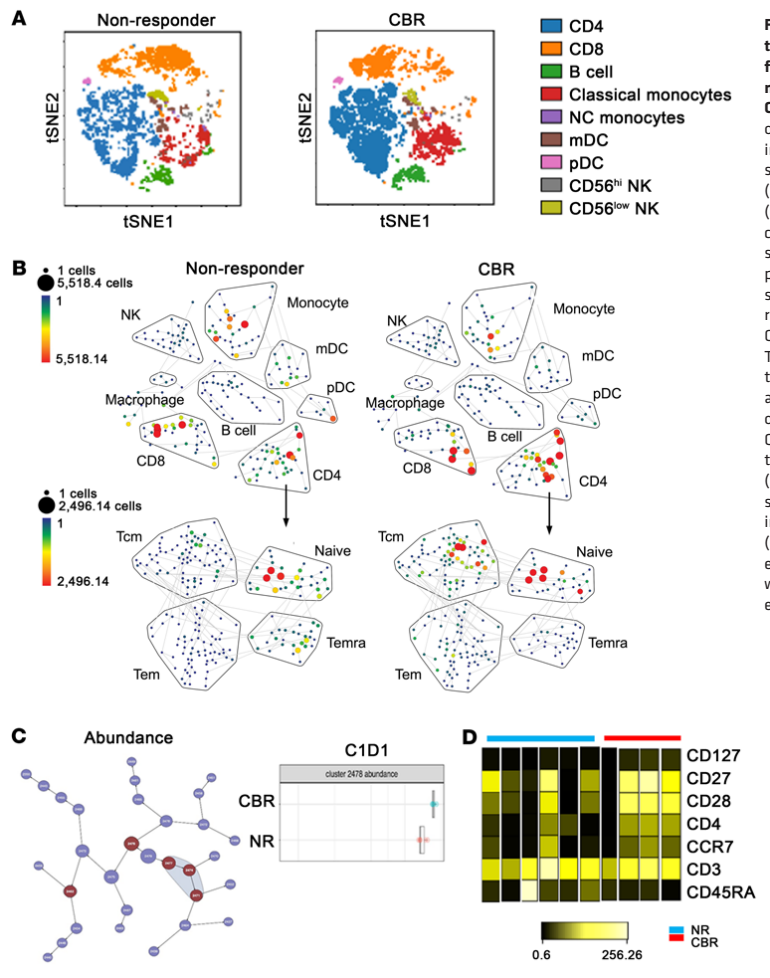
Mass spectrometric identification of differences in subsets of PBMCs from patients with a durable CBR and nonresponders before treatment with G+P. (**A**) Exemplified *t*SNE visualization of overlaid cell population composition in PBMCs from an initial cohort of nonresponders (*n =* 6) and durable CBR patients (*n =* 4) before the initiation of therapy (C1D1). (**B**) SPADE analysis of total immune cell populations in PBMCs from nonresponders (upper left) and durable CBR patients (upper right); as well as the subsets among total CD4^+^ T cells from nonresponders (lower left, *n =* 6) and durable CBR patients (lower right, *n =* 4) at C1D1. The size and color of each node correspond to the number of cells. (**C**) Left: CITRUS analysis showing a visual representation of unsupervised hierarchical clustering of CD4 cells and visualization of the clusters that were part of the significant results (in red). Right: Abundance plots for the significant cluster 2478 (of CD4 cells) in nonresponders (NR) and responders (CBR). (**D**) Heatmap represents the median expression levels of indicated markers within the cluster 2478 from nonresponders and responders (CBR).

We conducted the initial high-dimensional mass cytometry by time-of-flight (CyTOF) analysis with 20 cryopreserved PBMC samples isolated from the blood of a cohort of 10 patients before (C1D1) and after (C2D5) treatment. We used the dimensionality reduction tool viSNE to compare durable CBR patients with nonresponders prior to combination therapy. Live, intact single cells gated from the C1D1 PBMCs could be clearly grouped into distinct subsets (Figure 3A), including CD4^+^ T cells (CD3^+^CD4^+^), CD8^+^ T cells (CD3^+^CD8^+^), B cells (CD19^+^CD20^+^), classic monocytes (Lin^–^CD14^+^CD16^–^), nonclassical (NC) monocytes (Lin^–^CD14^lo^CD16^+^), monocytic DCs (mDCs), plasmacytoid DCs (pDCs), CD56^hi^ and CD56^dim^ NK cells (CD3^–^CD56^+^CD16^+/lo^) on the viSNE map. The gating strategy is shown in Supplemental Figure 3. Although the CD4^+^ T cells tended to be enriched in responders on the viSNE map, the frequencies of all baseline major immune subsets using conventional supervised gating in FlowJo did not show significant differences between durable CBR patients and nonresponders, as defined above (Supplemental Figure 4A). Given the key role of T cells in response to anti–PD-1 immune checkpoint blockade therapy, we focused on this cell type. We examined both CD4^+^ and CD8^+^ T cell populations using the combination of CD45RA, CD27, CD28, and CCR7 cell-surface markers to define naive (N) (CD45RA^+^CD27^+^CD28^+^CCR7^+^), central memory (CM) (CD45RA^−^CD27^+^CD28^+^CCR7^+^), effector memory (EM) (CD45RA^−^CD27^+/−^, CD28^+/−^, CCR7^−^), and terminally differentiated effector memory (EMRA) (CD45RA^+^CD27^−^CD28^−^CCR7^−^) cells. We found no significant differences in these T cell memory subsets between patients with a durable CBR and nonresponders (Supplemental Figure 4B).

To minimize investigator-associated biases and variability in the results inherent to supervised manual analysis of cytometric data, we used a minimally supervised, standardized analytical workflow based on the spanning-tree progression analysis of density-normalized events (SPADE) algorithm, in complement to conventional manual gating and supervised analysis. Live, intact single cells were clustered using cell-surface markers into a SPADE tree that identified major immune cell subsets, and further clustered among CD4^+^ T cell populations. Interestingly, we observed different abundance patterns of subclusters within CD4^+^ N and CM populations between durable CBR patients and nonresponders (Figure 3B). Closer examination by CITRUS — a clustering-based supervised algorithm that identifies stratifying clusters — revealed a higher frequency of Cluster-2478 in the T cell compartment of patients with a durable CBR compared with that of nonresponders (Figure 3C). A heatmap of expression of CD4^+^ T cell memory surface markers further showed that this cluster was positive for CCR7, CD27, and CD28, with dim or null expression of CD45RA and CD127 (Figure 3D), indicating a mixture of CD4^+^ N and CM subsets. These results suggest that baseline CD4^+^ N and/or CM T cells may be predictive biomarkers of a response to guadecitabine plus pembrolizumab (G+P).

**Figure 4:**
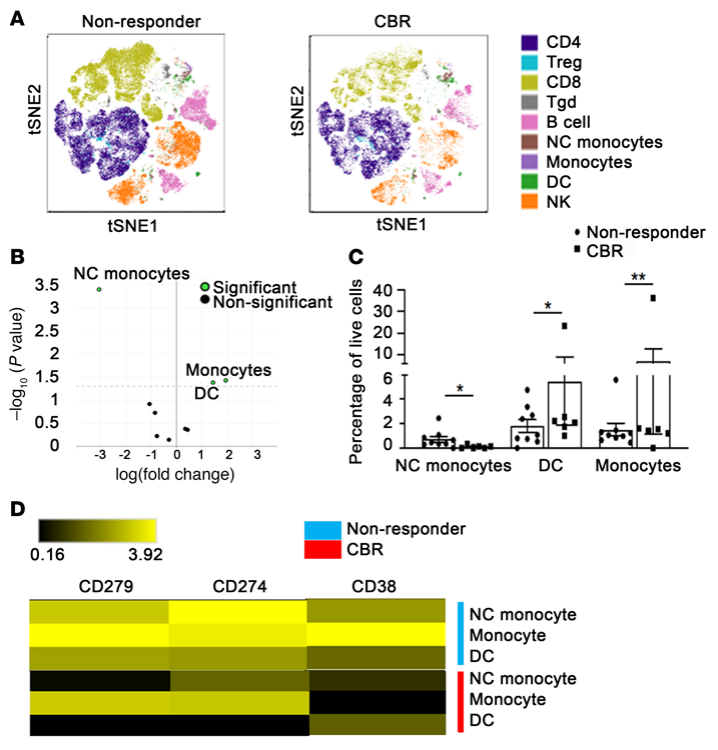
Differences in the frequencies of myeloid cell populations between patients with a durable CBR and nonresponders before treatment with G+P, by spectral cytometry. (**A**) Exemplified *t*SNE visualization of the overlaid cell population composition in PBMCs from an extended cohort of nonresponders (*n =* 9) and patients with durable CBRs (*n =* 6) before the initiation of therapy (C1D1). (**B**) edgeR analysis identified myeloid cellular populations with significant differences in relative abundance between nonresponders and patients with a durable CBR. (**C**) Differences in the percentages of NC monocytes, DCs, and classical monocytes among live PBMCs from nonresponders or durable CBR patients at C1D1. Data indicate the mean ± standard deviation. *P < 0.05 and **P < 0.01, by 2-tailed *t* test with multiple-comparison correction using the Benjamini-Hochberg adjustment. (**D**) Heatmap represents the median expression for markers (CD279, CD274, and CD38) that were differentially expressed (adjusted *P <* 0.05) in NC monocytes, DCs, and classical monocytes between nonresponders and durable CBR patients.

**Figure 5:**
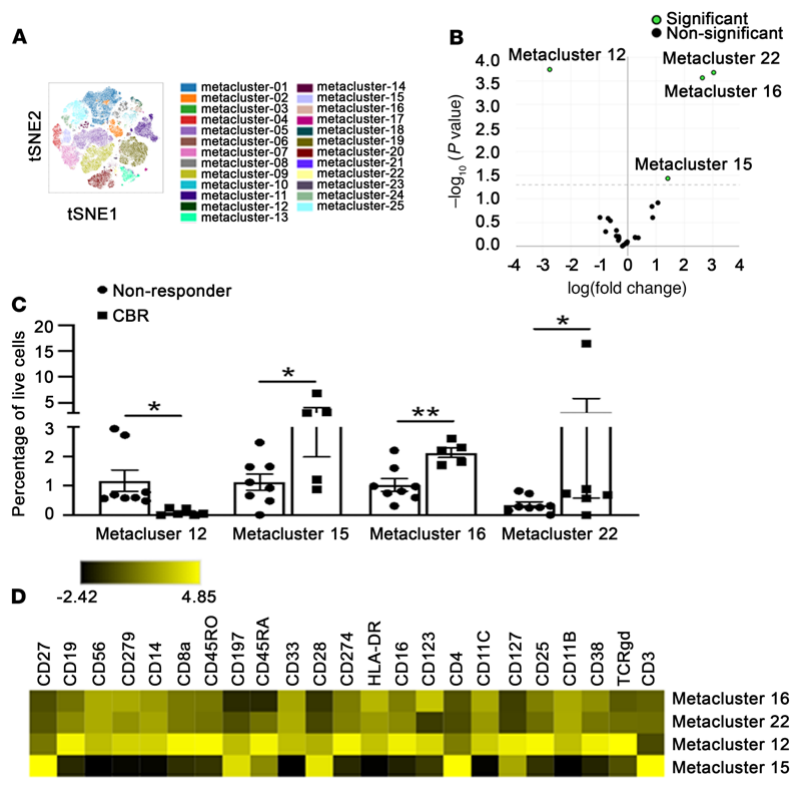
Identification of metaclusters in PBMCs with a significant difference between patients with a durable CBR and nonresponders before treatment with G+P. (**A**) Exemplified *t*SNE visualization of overlaid unsupervised metaclusters in PBMCs using the FlowSOM algorithm from an extended cohort of nonresponders (*n =* 9) and patients with a durable CBR (*n =* 6) at C1D1, prior to therapy. (**B**) edgeR analysis identified metaclusters with significant differences in relative abundance between nonresponders and patients with a durable CBR. (**C**) Differences in the percentages of unsupervised metaclusters in PBMCs from nonresponders and patients with a durable CBR at C1D1. Data indicate the mean ± standard deviation. *P < 0.05 and **P < 0.01, by 2-tailed *t* test with multiple-comparison correction using the Benjamini-Hochberg adjustment. (**D**) Heatmap represents the median expression levels of the indicated markers within the metaclusters that had significant differences in relative abundance between nonresponders and patients with a durable CBR.

As the classic monocytes tended to be more frequent in pretreatment specimens from patients with a durable CBR before therapy, we examined in depth the myeloid cellular components in an extended cohort of 15 patients, which included 6 patients with durable CBRs and 9 nonresponders (pretreatment specimens). The PBMCs were characterized using a 27-color, high-dimensional spectral flow cytometry (CyTEK) panel that incorporated hallmark surface markers for all major immune cell populations, comparable to the CyTOF panel used above (Figure 4A). The differential abundance of all baseline major immune cell subsets based on conventional supervised gating was analyzed using the edgeR package (Figure 4B). Despite the relatively high variability within each group, we found a statistically greater abundance of classical monocytes and DCs in patients with durable CBRs but a statistically greater abundance of NC monocytes in nonresponders prior to therapy (Figure 4C). When we examined in detail the functional phenotypes of these cell populations that were differentially abundant, we found that nonresponders had higher expression levels of PD-1 (CD279), PD-L1 (CD274), and CD38 in both classical and NC monocyte compartments than did responders (Figure 4D). Moreover, increased expression levels of PD-1 and PD-L1 were observed in DCs of nonresponders (Figure 4D), which could be indicative of the stronger immunomodulatory properties of these myeloid cell subsets in nonresponders reported during immunotherapy. We next assessed the changes in abundance of all major immune cell subsets and found a lower abundance of classical monocytes and DCs after therapy as compared with baseline values in responders (Supplemental Figure 5). For unsupervised clustering to yield 25 metaclusters in PBMCs prior to therapy, we used the FlowSOM algorithm (Supplemental Figure 6) and the dimensionality reduction tool viSNE (Figure 5A). edgeR analysis (Figure 5B) identified an increase in clusters 16 and 22 (representing CD14^+^CD16^lo^ intermediate and/or classical monocyte populations that express HLA-DR, CD33, CD11b, and CD11c) as well as cluster 15 (containing CD45RA^+^CD27^+^CD28^+^CCR7^+^ CD4 N-like T cells) in patients with a durable CBR compared with nonresponders (Figure 5, C and D). By contrast, this clustering approach identified an increase in CD19^+^CD38^+^CD27^–^ immature-like B cells (cluster 12) in nonresponders compared with patients with a durable CBR before therapy (Figure 5, C and D). Collectively, the results of this multidimensional analysis based on several analytic approaches indicate an enrichment of distinct circulating populations of CD4 N-like T cells and monocytes in responders at baseline.

To determine therapy-induced antitumor immune responses in the TME, we examined the immune profiles in the malignant ascites of 1 individual patient with a durable CBR using CyTOF analysis. Projecting the CyTOF data for major immune subsets in *t*-distributed stochastic neighbor embedding (*t*SNE) space revealed a relative enrichment in CD4^+^ and CD8^+^ T cells, B cells, and NC monocyte populations in post-treatment ascites compared with pretreatment ascites, whereas we observed a lower accumulation of classical monocyte populations in post-treatment ascites compared with pretreatment ascites (Supplemental Figure 7A). The monocyte phenotype switch was dominated by increased CD16 expression among the CD14^+^ monocyte population in ascites in response to therapy (Supplemental Figure 7B). Moreover, a greater number of T cells from post-treatment ascites produced IFN-γ in response to anti-CD3 (Supplemental Figure 6C, left) or the HLA-A2–binding peptide NY-ESO-1 (Supplemental Figure 7C, right), compared with pretreatment ascites.

**Figure 6:**
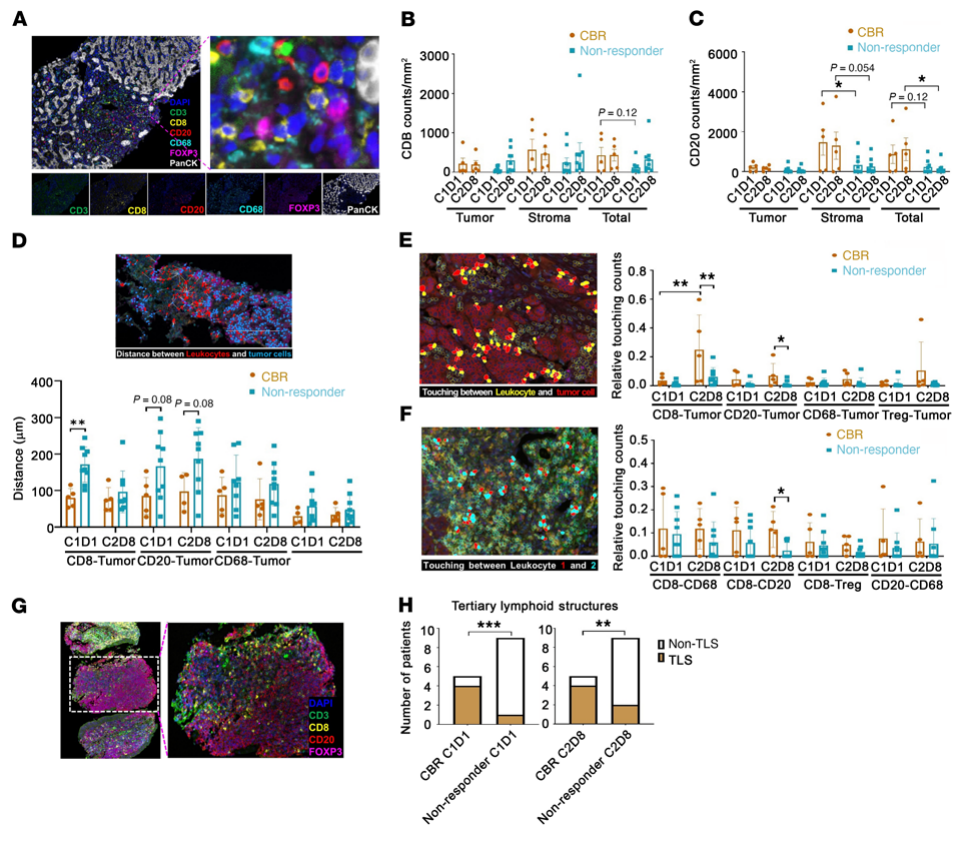
Distinct features of TILs between nonresponders and patients with a durable CBR. (**A**) Representative multiplex image (upper left panel) with inset (upper right) of cytotoxic T cells (CD3^+^CD8^+^), B cells (CD20^+^), macrophages (CD68^+^), and Tregs (CD3^+^CD8^–^FOXP3^+^) in a responder patient after treatment with G+P, measured by mIHC. (**B** and **C**) Density of tumor-infiltrating CD8^+^ T cells (**B**) and CD20^+^ B cells (**C**) in the compartments of the tumor nest and stroma from patients with a durable CBR (*n =* 5) and nonresponders (*n =* 9). (**D**–**F**) Spatial characterization among TILs and tumor cells from patients with a durable CBR (*n =* 5) and nonresponders (*n =* 9). (**D**) Distance from TILs (red) to tumor cells (blue). (**E**) Touching events between TILs (yellow) and tumor cells (red). (**F**) Touching events among TILs. (**G**) Representative image of mIHC staining of putative TLSs in a patient with a durable CBR after treatment with G+P. (**H**) Comparison of putative TLSs at C1D1 (left) and C2D8 (right) between patients with a durable CBR (*n =* 5) and nonresponders (*n =* 9). Box and whiskers represent the mean ± standard deviation, and each dot represents 1 patient. Original magnification, ×40 (**A** and **D**–**G**). Higher-magnification images in and **A** and **G** were generated in Photoshop by selecting the indicated areas using the crop tool, and then expanding the areas. *P < 0.05, **P < 0.01, and ***P < 0.001, by 2-way ANOVA followed by multiple-comparison correction (**B**–**F**) and the Mann-Whitney *U* test (**H**).

To gain a deeper understanding of potential mechanisms of therapeutic immune responses, we examined paired pre- and post-treated tumor tissue sections for immune infiltration by multiplex IHC (mIHC) using Opal staining, which allowed for the simultaneous assessment of 7 markers in a single formalin-fixed, paraffin-embedded (FFPE) tissue section. We initially focused on the following set of markers: pancytokeratin (PanCK, epithelial marker), CD3, CD8, FOXP3, CD68, and DAPI (nuclear stain) (Figure 6A). Through the spectral unmixing algorithm, we found that the baseline samples tended to display a greater density of CD8^+^ T cells (Figure 6B) and CD20^+^ B cells (Figure 6C) in the total analyzed areas, although statistical significance was not reached. By contrast, there were no significant differences in the density of either CD68^+^ macrophages (Supplemental Figure 8A) or FOXP3^+^ Tregs (Supplemental Figure 8B) between C1D1 and C2D8 specimens or between the tumor nest and stromal area of tissue sections from patients with a durable CBR and nonresponders, respectively. Notably, we found a significantly higher density of CD20^+^ B cells in the stromal compartment in samples from patients with a durable CBR than in those from nonresponders, particularly in baseline samples (Figure 6B). The density of CD20^+^ B cells in the total analyzed area, including both the tumor nest and stromal compartment, was also significantly higher in post-treatment samples from patients with a durable CBR than those from nonresponders (Figure 6C).

We analyzed the distances between cells within the tumor core for their interaction and identified CD20^+^ B cells as the major population located in proximity to tumor cells, a cell population that tended to be closer to tumor cells in patients with a durable CBR than in nonresponders (Figure 6D). The subsequent spatial analysis revealed that either CD8^+^ T cells or CD20^+^ B cells were more likely to be touching tumor cells in C1D1 and C2D8 samples from patients with a durable CBR compared with those from nonresponders (Figure 6E). In addition, there was a greater abundance of CD8^+^ T cells touching CD20^+^ B cells in the post-treatment samples from durable CBR patients than in samples from nonresponders (Figure 6F). Quantification showed that there was no significant touching of other immune cells with tumor cells in the C1D1 and C2D8 samples from patients with a durable CBR and nonresponders, respectively. Interestingly, architectural analysis indicated that CD20^+^ B cells were localized in putative tertiary lymphoid structures (TLSs) of tumors from patients with a durable CBR and were colocalized with CD3^+^, CD8^+^, and FOXP3^+^ T cells (Figure 6G). We found that 4 of 5 pre- or post-treatment samples from patients with a durable CBR exhibited one or more TLSs, whereas only 1 or 2 of 9 pre- or post-treatment samples from nonresponders had TLSs (Figure 6H).

**Figure 7:**
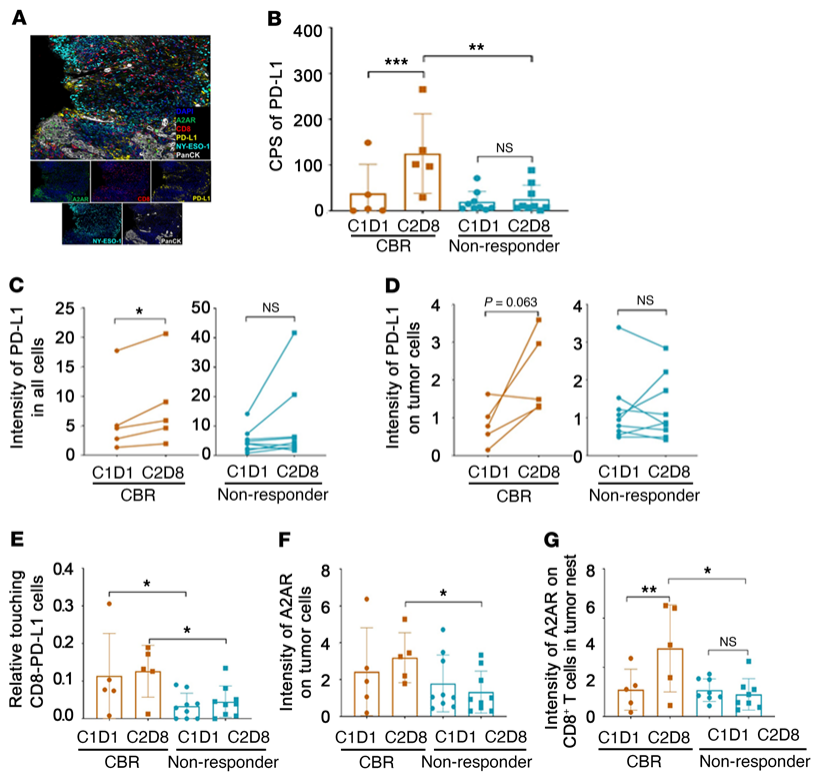
Increased expression levels of PD-L1 and A2AR within tumors related to a favorable clinical response to G+P treatment. (**A**) Representative multiplex (upper panel) and singlet immunostaining (lower panels) images for the following markers: PD-L1, NY-ESO-1, A2AR, CD8, PanCK, and DAPI. Original magnification, ×40. (**B**) Comparison of PD-L1 expression in tumors by combined positive score (CPS) between patients with a durable CBR (*n =* 5) and nonresponders (*n =* 9). (**C** and **D**) Expression levels of PD-L1 on total cells (**C**) and on PanCK^+^ tumor cells (**D**) at C1D1 and C2D8 between patients with a durable CBR (*n =* 5) and nonresponders (*n =* 9). (**E**) Touching events between PD-L1^+^ cells and CD8^+^ cells at C1D1 and C2D8 in cells from patients with a durable CBR (*n =* 5) and nonresponders (*n =* 9). (**F** and **G**) Expression levels of A2AR on tumor cells (**F**) or on tumor nest–infiltrating CD8^+^ T cells (**G**) at C1D1 and C2D8 between patients with a durable CBR (*n =* 5) and nonresponders (*n =* 9). Box and whiskers represent the mean ± standard deviation, and each point represents 1 patient. *P < 0.05, **P < 0.01, and ***P < 0.001, by 2-way ANOVA with multiple-comparison correction (**B** and **E**–**G**), and 2-tailed paired *t* test (**C** and **D**).

We ran a second mIHC panel to assess differences in protein levels of PD-L1, adenosine A2A receptor (A2AR), and NY-ESO-1 in CD8^+^ T cells and tumor cells (Figure 7A) in patients with a durable CBR versus nonresponders, before and after treatment. We detected a significantly higher density of PD-L1^+^ cells (Figure 7B) and significantly higher levels of PD-L1 expression (Figure 7C) in all cells (tumor and nontumor) in post-treatment versus baseline samples from patients with a durable CBR, but not in those from nonresponders. Similarly, the post-treatment specimens from patients with a durable CBR tended to show increased expression levels of PD-L1 in tumor cells compared with baseline samples, but not in tumor cells from nonresponders (Figure 7D). Furthermore, CD8^+^ T cells were more likely to be touching PD-L1^+^ cells in both baseline and post-treatment samples from patients with a durable CBR compared with samples from nonresponders (Figure 7E). We also observed reduced A2AR intensity in both tumor (Figure 7F) and CD8^+^ T cells (Figure 7G) in post-treatment samples from patients with a durable CBR compared with nonresponders. A2AR intensity on CD8^+^ T cells in the tumor nest was significantly elevated in post-treatment samples compared with baseline samples from patients with a durable CBR, but not in those from nonresponders (Figure 7G). In contrast, we did not observe significant differences in NY-ESO-1 intensities in tumor cells between the baseline and post-treatment samples from patients with a durable CBR and nonresponders, respectively (Supplemental Figure 8C).

Last, changes in 7 inflammatory cytokines were examined in relation to responses to G+P. Notably, we found that IL-8 levels were significantly increased in nonresponders but decreased in patients with a durable CBR upon progression (C3D1 vs. 30 days after discharge from the study) (Supplemental Figure 9). Similarly, IL-6 levels were significantly decreased in patients with a durable CBR upon progression (C3D1 vs. 30 days after discharge from the study), while the levels tended to remain elevated in nonresponders (Supplemental Figure 9). Increased levels of TNF-α were also observed in nonresponders but not in patients with a durable CBR upon progression (C3D1 vs. 30 days after discharge from the study) (Supplemental Figure 8). The results indicate that changes in serum IL-8 and IL-6 levels may be associated with a response to G+P therapy in patients with OC, consistent with data showing that baseline levels of these cytokines predict shorter survival times and reduced clinical benefit from PD-1/PD-L1 checkpoint blockade in other solid tumors (23–26).

## Discussion

ICIs have yet to make an impact in OC, either as single agents or in combination with chemotherapy (2, 5). Understanding their effects in vivo could lead to improved treatment responses, either by identifying ways to select patients likely to benefit or by providing the rationale for new drug combinations. There are no current markers predictive of response or resistance to ICIs in patients with OC. Here, we propose that the presence of N, CM CD4^+^ T cells and activated classical monocytes in the circulation, as well as a greater density of CD8^+^ T cells and CD20^+^ B cells and detection of TLSs in tumor tissue predispose and allow patients to benefit from immune interventions. We dissected the effects of this regimen on tumor-immune cell interactions relative to clinical benefit.

Although this phase II study combining epigenetic priming with an ICI did not demonstrate sufficient clinical activity to advance this regimen for further development, detailed analyses of the immune environment in enrolled patients provide new insights into how and in whom this combination elicits antitumor immunity. Notably, the combination was well tolerated clinically, with no worrisome signals when compared with the known toxicity profiles of ICIs or guadecitabine, and the 32% clinical benefit rate highlights that selected patients derive sustained benefit.

This trial used a lower cumulative dose of guadecitabine per cycle compared with the doses used in previous leukemia and OC clinical trials (27–30), however, modest, but significant, post-treatment global DNA hypomethylation was observed in both PBMCs and tumors, showing that the regimen achieved its intended biological effects. Pathways enriched in genes associated with significantly demethylated CpG sites included those for Notch, Wnt, and TGF-β signaling, which had been identified in other studies as being reactivated in response to HMAs (18, 31, 32). In concert with the methylation changes induced by this combination, we observed robust transcriptomic effects in tumor biopsies. Importantly, gene networks related to immune responses were highly enriched after treatment, with the top upregulated transcripts being those for the cytokines *IFNG*, *CXCL9*, *IL21*, and several members of the granzyme family, as well as for the TA *PNMA5*, indicating robust activation of antitumor immunity in vivo. These results are comparable, but more pronounced, compared with observations from previous tumor analyses in studies using HMAs and carboplatin to treat women with recurrent OC (17, 18, 31), which had indicated that pathways related to immunity are reactivated by DNA hypomethylation. Reexpression of endogenous retroviral elements integrated into the genome and leading to an IFN response has been detected in cancer cells exposed to HMAs (7). Additionally, we show in vivo activation of antigen-specific (NY-ESO-1) cytotoxic T cells in the cell compartment isolated from 1 responding patient’s malignant ascites. In contrast, upregulation of transcripts with known immune-suppressive roles, such as *IDO1,* encoding the enzyme indoleamine 2,3-dioxygenase 1, and the T cell receptor *Lag3,* with immune checkpoint function. Upregulation of these transcripts possibly reflects the effects of the HMA used in this regimen, as both genes are regulated by promoter methylation (33–35), and their upregulation could act as a break in the immune response elicited by treatment. These observations also fuel the hypothesis that inclusion of an additional blocking strategy could be necessary to overcome the effects of *LAG3* or *IDO1* upregulation by the HMA in this regimen and that a 3-drug combination could enhance efficacy.

We used clustering to define immune cell populations implicated in antitumor response and found a higher frequency of subsets of peripheral N and/or CM CD4^+^ T cells in responders before therapy. Our findings support the value of baseline CD4^+^ memory T cell quantification to predict the efficacy of PD-L1/PD-1 inhibitors. This is consistent with recent studies (36–38) showing the importance of peripheral CD4^+^ memory T cell subsets before the start of immunotherapies with predictive capacities for clinical benefit. Thus, it is likely that systemic CD4 immunity might be required to achieve effective CD8 responses upon PD-L1/PD-1 blockade therapy.

Besides a modest change in the CD4^+^ memory T cell subsets before therapy, we suggest a role of the baseline frequency of classical monocytes in discriminating patients with distinct clinical outcomes, similar to what has been described for the predictive value of the frequency of peripheral CD14^+^CD16^−^CD33^+^HLA-DR^hi^ monocytes in anti–PD-1 blockade therapy for patients with melanoma (39). Moreover, the lower levels of PD-L1/PD-1 on these monocytes from responders before therapy are likely associated with fewer immunosuppressive features, thus facilitating the development of an effective peripheral antitumor CD8 immune response in concert with CD4^+^ memory T cells during anti–PD-1 immunotherapy. This is also consistent with our observations in post-treatment ascites from 1 individual responding patient that revealed a relative enrichment in CD4^+^ and CD8^+^ T cells, B cells, and monocyte populations with enhanced TA-specific T cell responses, further supporting a role of CD4^+^ T cells and monocytes in antitumor immune responses upon PD-L1/PD-1 blockade therapy.

Consistent with the notion that the presence of CM CD4^+^ T cells and activated classical monocytes may be required for a successful antitumor response upon anti–PD-1 immunotherapy, we report a greater density of CD8^+^ T cells and CD20^+^ B cells in pretreated tumor tissues from patients with durable CBRs than in those from nonresponders. While intratumoral B cells are a multifaceted subset that have both pro- and antitumoral roles (40), emerging evidence demonstrates that the presence of B cells and TLSs is correlated with a favorable response to ICI in patients with metastatic melanoma (41, 42), renal cell carcinoma (42), or soft tissue sarcomas (43). Indeed, we found that CD20^+^ B cells were localized in putative TLSs within tumors and that the CD8^+^ T cells touching CD20^+^ B cells as well as tumor cells were more abundant in the pre- and post-treatment samples from patients with a durable CBR than in samples from nonresponders, supporting a potential role of intratumoral B cells and TLSs in promoting a T cell–mediated ICI response. This is corroborated by our observation that patients with a durable CBR were more likely to harbor TLSs in response to ICI compared with nonresponders. It should be noted, however, that although the overall presence of intratumoral B cells and TLSs had a marked impact on clinical benefit, their functional status was not measured and remains elusive. It is currently unknown why some patients develop organized TLSs while others do not. It has been speculated that TLSs may be directly activated by ICI treatment to augment antitumor effects potentially via expansion of incoming T cells (44) as well as sustainment of B cell maturation and antibody production (45). Combining our panel with additional multiplex panels for functional markers of T cell activation, as well as markers of additional components of the TLS, such as B cells and follicular DCs, may elucidate whether an active cooperation of these cell subsets elicits successful immunotherapy responsiveness.

We also detected the upregulation of PD-L1 on total cells including tumor cells from patients with a durable CBR rather than from nonresponders during anti–PD-1 therapy, and we observed a greater abundance of CD8^+^ T cells that were touching PD-L1^+^ cells in patients with a durable clinical benefit rate. This is likely the result of an effective immune response against tumors and elevated levels of IFN-γ, which directly induces PD-L1 expression (46). In recent years, the immunosuppressive role of the CD73/A2AR axis in cancer has been investigated (47, 48), and the results suggest it may represent another attractive target complementing PD-1/PD-L1 and CTLA-4 blockade for antitumor immunotherapy (49–51). As with PD-L1, we found upregulated A2AR expression on CD8^+^ T cells from patients with a durable CBR rather than in those from nonresponders during anti–PD-1 therapy, suggesting a potential compensation between PD-1/PD-L1– and A2AR-mediated immune suppression that has been described previously in preclinical studies (49). Although the key mechanism underlying this upregulation of A2AR expression is not known, it is possible that treatment with a HMA led to its upregulation, given previous reports showing that promoter methylation plays a role in the expression of A2AR (52, 53). Our results support further evaluation of the use of A2AR inhibitors in combination with current ICI therapy, especially among the cohort of patients with refractory OC.

In all, our study provides an in-depth characterization of the immune response to epigenetic priming plus ICI treatment in patients with chemotherapy-resistant OC, proposes new features that are predictive of benefit, and suggests potential barriers to achieving clinical success. Advancement of immunotherapy strategies in OC will require consideration of the previously underappreciated role of B cells, which may need to be co-opted in synergistic interventions, or the addition of other immune modulators, such as those targeting *LAG3*, *IDO1*, and *A2AR*, which may exert unanticipated inhibitory responses.

## Methods

### Study design and participants.

This was a nonrandomized, open-label, 2-stage phase II clinical trial performed at 3 sites to test the combination of G+P therapy in recurrent platinum-resistant OC. Patients had epithelial ovarian, fallopian tube, or primary peritoneal cancer that had recurred or progressed less than 6 months after their last dose of platinum-based chemotherapy. Other key eligibility requirements are described in the Supplemental Methods. The study was registered with ClinicalTrials.gov (NCT02901899).

### Procedures.

Guadecitabine (30 mg/m^2^) was administered by s.c. injection on days 1–4 of a 21-day cycle. On day 5, pembrolizumab (200 mg) was administered i.v. Each cycle was 21 days (Figure 1A). The drug combination was given until progression of disease or unacceptable toxicity. Imaging-guided tumor biopsies, ascites, or blood for the determination of cytokine responses, and PBMCs were obtained from consenting patients at specific time points (see Supplemental Methods and Figure 1A). Three 18 gauge tumor cores were obtained on C1D1 and C2D8, verified by a board-certified pathologist to contain greater than 50% tumor content, and immediately snap-frozen (~25–50 mg/specimen). When available, ascites or pleural fluid was centrifuged, and fluid and cell pellets were separated prior to cryopreservation.

### Outcomes.

The primary outcome of the trial was an objective response rate (ORR), defined as the proportion of patients with a CR or a PR using RECIST, version 1.1. Secondary objectives included PFS, a CBR defined as the proportion of patients with an ORR or SD for at least 3 months, and toxicity. Any patient who received at least 2 cycles of treatment was evaluable for the ORR. Patients with a durable clinical benefit were defined as any patient who experienced clinical benefit and received at least 6 cycles of treatment (CBR). Toxicity was classified according to CTCAE, version 4.03, and categorized as unrelated or possibly, probably, or definitely related to each study drug (see the Supplemental Materials for details). Translational endpoints were LINE-1 methylation in DNA obtained from PBMCs, global tumor methylation before and after treatment, and analysis of tumor-infiltrating leukocytes (TILs) in tumor biopsies before and after treatment.

### DNA and RNA extraction.

DNA and total RNA were extracted from PBMCs and tumor biopsies using the AllPrep DNA/RNA/Protein Mini Kit (QIAGEN). DNA and RNA concentrations were measured with absorbance set at 260 nm, and purity was estimated by calculating the 260:280 nm absorbance ratio.

### DNA methylation analysis by pyrosequencing.

Methylation levels of LINE1 in PBMCs were measured by bisulfite pyrosequencing at EpigenDx as described previously (54).

### Methylome analysis.

Methylation levels of over 850,000 CpGs in paired tissue samples (C2D8 vs. C1D1) from 11 patients were measured using the Infinium Human MethylationEPIC Beadchip array (Illumina). DNA (500 ng) was bisulfite converted and used for methylation profiling at the NUSeq Core Facility of Northwestern University, according to the Illumina’s protocol (see Supplemental Methods).

### Transcriptome analysis.

RNA-Seq libraries were prepared with a NEBNext Ultra II RNA Library Preparation Kit (New England BioLabs), as described in the manufacturer’s protocol and in Supplemental Methods, and were sequenced at the NUSeq Core Facility of Northwestern University.

### Sample staining and data acquisition for CyTOF.

Sample staining was performed for CyTOF analysis as described previously (55). In brief, cryopreserved cells were thawed and incubated for 10 minutes in prewarmed complete RPMI 1640 (RPMI, 10% FBS, penicillin and streptomycin). After washing with PBS, cells were incubated for 5 minutes at room temperature in 200 μL of 1 μM cisplatin solution (Fluidigm) for viability staining. Cisplatin was quenched by adding 2 mL of 5% serum-containing PBS. Following the washing with FACS buffer, cells were stained with a metal-conjugated surface stain antibody cocktail for 20–30 minutes at 4°C. Cells were then washed, filtered, and resuspended in MilliQ water for data acquisition on a Helios-upgraded CyTOF 2 mass cytometer (Fluidigm) through the Human Immune Monitoring Center (HIMC) at Stanford University. The metal-conjugated antibodies used (Supplemental Table 9) were either purchased from Fluidigm or conjugated in-house using MaxPar X8 reagent kits (Fluidigm) obtained from the HIMC, according to the manufacturer’s protocol.

### Validation by CyTEK.

Validation of the CyTOF data was conducted by detection of similar markers with a fluorescence-conjugated antibody cocktail using a CyTEK Aurora full-spectrum flow cytometer (Cytek Biosciences) as described previously (56) through the Immunotherapy Assessment Core at Northwestern University. These fluorescence-conjugated antibodies were purchased from BioLegend and BD Biosciences and are listed in Supplemental Table 10.

### Cytometric data quantification and analysis.

Analysis of CyTOF and CyTEK data was performed as previously described (56). Briefly, the FCS files generated were manually gated to live CD45^+^ cells, downsampled, and sequentially gated for the merged data sets using FlowJo software (BD). Clustering analyses were performed using the viSNE (57), FlowSOM (58), SPADE (59), and CITRUS (60) algorithms within the Cytobank and OMIQ web applications according to the developers’ instructions. All events were sampled with a minimum estimated cluster size of 1% (~1000 events). The Significance Analysis of Microarrays (SAM) association model (61) was used for clustering analysis. For the differential analyses, we used the edgeR method (62). Select significant FlowSOM clusters were plotted onto the viSNE map for visualization.

### Multiplex cytokine assays.

For the plasma cytokine detection studies, the Human ProInflammatory 7 Ultra-Sensitive Kit from Meso Scale Diagnostics (MSD), which measures IFN-γ, IL-6, IL-8, IL-10, TNF-α, IL-12p70, and IL-1β, was used. MSD plates were analyzed on MSD’s MS2400 imager according to the manufacturer’s instructions. All standards and samples were measured in duplicate.

### mIHC.

The formalin-fixed, paraffin-embedded (FFPE) tumor biopsies obtained before (C1D1) and after (C2D8) treatment were analyzed by mIHC staining using the Opal 7-Color Multiplex IHC kit (Akoya Biosciences) as described previously (63). Briefly, 5 μm FFPE tissue sections were deparaffinized, rehydrated, and refixed with 10% neutral buffered formalin prior to antigen recovery in heated AR9 retrieval buffer (Akoya Biosciences) for 15 minutes. Afterwards, the FFPE sections underwent 6 sequential cycles of staining procedures. Each cycle included blocking, binding of the primary antibody and the corresponding HRP-labeled secondary antibody, and then visualized by a different Opal fluorophore. Each cycle was ended with another heated antigen retrieval process with AR6 retrieval buffer to remove the bound antibody. After the 6-cycle staining procedures, the sections were counterstained with DAPI (Akoya Biosciences) and mounted with Diamond Antifade fluorescence mounting media (Thermo Fisher Scientific). Each single marker with an associated fluorophore staining section served as a reference control in the spectral library for the “spectral unmixing process,” and the unstained slide served as the background control. Each biopsy was used for 2 panels of mIHC staining with the antibodies and corresponding fluorophores listed in Supplemental Table 11.

### Acquisition of multispectral images and data analysis.

The stained sections were imaged using the Vectra 3 Automated Quantitative Pathology Imaging System (PerkinElmer) equipped with DAPI, FITC, Cy3, Texas Red, and Cy5 emission spectral filter cubes. Images were acquired by scanning the whole slide at low magnification (×4), from which multiple regions of interest (ROI) on each section that included adequate distribution of different markers were captured at high magnification (×20) through the 5 emission spectral filters. The single fluorophore staining section was also obtained through the same imaging protocol. The raw ROI images underwent a spectral unmixing process yielding 7 individual fluorophores based on the unique emission spectral pattern of each single staining fluorophore, using InForm Advanced Image Analysis software (Akoya Biosciences). Subsequently, the unmixed images were processed using the proprietary InForm active learning algorithm, including tissue segmentation into tumor nest and stroma based on individual cell-specific markers and a DAPI nuclear counterstaining marker, all of which were associated with specific *x*- and *y*-axis spatial coordinates. Analysis was implemented with the same algorithm to maintain consistency across all samples. The data from composite images, tissue segmentation, cell segmentation, and cell phenotyping from InForm were exported for further analyses of cellular densities, protein intensities, cellular touching events, as well as distances between 2 different cell types among tumor nest and stromal compartments using R-based phenoptrReports and phenoptr (Akoya Biosciences).

### Data availability.

All high-throughput sequencing data and processed data have been deposited in the NCBI’s Gene Expression Omnibus (GEO) data repository (GEO GSE186825 and GSE188250). The analyses were performed using publicly available software as described in the Supplemental Methods.

### Statistics.

This was a single-arm trial in which Simon’s optimum 2-stage design was used to test the hypothesis that an ORR of 0.10 or less versus an ORR of 0.30 or higher would be significantly different with 90% power and a 0.05 type I error rate. Eighteen patients were to be enrolled in the first stage, and if at least 3 responses were observed, additional patients were to be enrolled, for a total of 35 patients. The null hypothesis would be rejected if 7 or more responses occurred among 35 evaluable patients. Participants who were not evaluable for responses could be replaced. An initial safety run-in cohort of 6 patients was used to confirm combination treatment safety, and the study was allowed to continue if 1 or fewer dose-limiting toxicity (DLT) events occurred within 5 weeks of the start of treatment (cycle 1 and 2 additional weeks; see Supplemental Methods). Comparison of values was performed using a 2-tailed *t* test, the Kruskal-Wallis test, or the Mann-Whitney *U* test for unpaired data; the Wilcoxon matched-pairs, signed-rank test for paired data via GraphPad Prism (GraphPad Software); and 2-way ANOVA followed by multiple-comparison correction with the Benjamini, Krieger, and Yekutieli method (64). Data are presented as the mean ± standard deviation unless otherwise stated.

### Study approval.

The study was approved by the IRB of Northwestern University, and all patients provided written informed consent prior to participation.

## Author contributions

DM and BZ conceptualized the study and acquired funding. HC, SC, PX, MC, GFF, JWM, EJT, and AP acquired data. MK, RK, RVD, PX, and SC analyzed the data. SC, PX, MC, HC, HH, and AMA conducted experiments. DM, BZ, HC, and MC wrote the original draft of the manuscript. DM, BZ, MK, and HC wrote, reviewed, and edited the manuscript. SC, PX, and MC share first authorship and are listed according to their relative contributions to the work.

## Supplementary Material

Supplemental data

Trial reporting checklists

ICMJE disclosure forms

## Version 1 06/07/2022

In-Press Preview

## Version 2 07/15/2022

Electronic publication
