# Supplementary material for: Epigenetic priming enhances antitumor immunity in platinum-resistant ovarian cancer
Source: J Clin Invest. 2022 Jul 15;132(14):e158800. doi: 10.1172/JCI158800 (PMC9282926; doi:10.1172/JCI158800)
Supplement: Supplemental data [file jci-132-158800-s066.pdf]

Supplementary Materials

Materials and Methods

Study design: Other eligibility criteria for the study included measurable disease by Response Evaluation Criteria in Solid Tumors 1.1, an Eastern Cooperative Oncology Group (ECOG) performance status of 0-1 and normal organ and marrow function. Patients were required to have at least one prior platinum-based therapeutic regimen, but those with more than three prior platinum-based regimens, or more than two non-platinum cytotoxic chemotherapy regimens were excluded. There was no limit on use of prior biological therapies, but prior immunotherapy was not allowed. Patients were also excluded if they had a diagnosis of immunodeficiency or were receiving systemic steroids or immunosuppressive therapy within 7 days of enrollment. An initial safety run-in cohort of 6 patients was used to confirm combination treatment safety, and the study was allowed to continue if <=1 dose-limiting toxicity (DLT) events occurred within 5 weeks of treatment start (cycle 1 and 2 additional weeks). DLTs were defined as any treatment-related grade $\geq$3 non-hematological toxicity occurring despite the use of adequate medical intervention and/or prophylaxis, grade 4 neutropenia lasting $\geq$ 7 days, grade 4 febrile neutropenia, grade 3 thrombocytopenia lasting $\geq$ 7 days, or grade 4 thrombocytopenia lasting $\geq$ 4 days, or any treatment-related toxicity that results in > 14 days delay in cycle 2 Day 1 dosing.

Procedures: Imaging-guided tumor biopsies, ascites, or blood for determining cytokine response and collection of PBMCs were obtained from consenting patients at baseline (cycle 1, day 1, C1D1) on day 5 of cycle 1 (C1D5), before the second cycle (C2D1), on day 5 of cycle 2 (C2D5), on day 8 of cycle 2 (C2D8), before cycle 3 (C3D1), and 30 days and 6 months after the end of

treatment, as shown in Figure 1A. Three 18-gauge tumor cores were obtained on C1D1 and C2D8 and the material was immediately snap frozen (~25-50mg/specimen) after pathological verification. When available, ascites or pleural fluid was centrifuged, and fluid and cell pellets were separated prior to cryo-preservation.

Outcomes: The primary outcome of the trial was objective response rate (ORR), equivalent to the total rate of complete response (CR) and partial response (PR). Secondary objectives included progression-free survival (PFS), clinical benefit rate (CBR) defined as the sum between ORR and total rate of stable disease (SD) at 3 months, and toxicity profiles. After a baseline scan, imaging modality remained constant per-patient throughout the study, and subsequent surveillance studies were obtained during treatment with every odd-numbered cycle starting with cycle 3. Response was assessed in measurable and non-measurable lesions using immune-related response criteria (irRC) and RECIST v1.1; progression was determined only by RECIST v1.1. Any patient who received at least two cycles of treatment was evaluable for ORR. Toxicity was monitored at each cycle by history, physical examination, measurement of serum electrolytes and indicators of renal, liver and marrow function. Beginning at cycle 3 and each subsequent cycle, thyroid function was also monitored. A safety follow-up visit occurred at 30 days after the last dose of study drug, or before the initiation of a new drug regimen. Adverse events related to study drugs were managed with dose withholding or delay; a delay of more than 28 days that was possibly, probably or definitely related to a study drug would result in withdrawal from study treatment.

Statistical design: The trial employed a Simon's two-stage design to test the null hypothesis that RR<=0.10 versus the alternative that RR>=0.30 (where RR is calculated via RECIST v1.1). The

design has an expected sample size of 22.5 and a probability of early termination of 0.71 when RR=0.10. If the drug combination was actually not effective, there was a 0.047 probability of concluding that it is (the target for this value was 0.050). If the regimen was actually effective, there was a 0.098 probability of concluding that it is not (the target for this value was 0.100). If three patients experienced clinical benefit in stage I [n = 18], enrollment proceeded to stage II with a total target enrollment of 35 evaluable patients.

Methylome analysis: DNA (500 ng) was bisulfite converted and used for methylation profiling at the NUSeq Core Facility, Northwestern University, according to the Illumina's protocol. BeadChips were scanned with an Illumina iScan and then analyzed using the Illumina GenomeStudio software. Probes were filtered based on quality of detection, bead count (< 3 in at least 5% of samples), lack of CpGs, SNP presence, and alignment to multiple locations. Sex chromosome probes were included, as all patients in the study were female. The resultant 742,725 probes were analyzed for differential methylation between post-treatment (C2D8) and baseline (C1D1) paired groups. Methylation analysis was performed using the ChAMP package. Methylation beta values were normalized by Beta MIxture Quantile dilation (BMIQ) (1). Batch effect correction was performed on Sentrix ID, via the ComBat package (2). Finally, a linear model was fitted for statistical testing between the two groups, using limma (via ChAMP) and associated t-test. The p-values were adjusted using the Benjamini-Hochberg procedure. Pathway analysis was performed using EnrichR.

Transcriptome analyses: RNA-seq libraries were prepared with a NEBNext Ultra II RNA library prep kit from Illumina (New England Biolabs Inc., Ipswich, MA). In brief, mRNA isolated from

1 mg of total RNA was used for first-strand cDNA synthesis, which was followed by second-strand cDNA synthesis, end-repair of cDNA library, dA-tailing of cDNA library, adaptor ligation, PCR enrichment, and verification of library quality with a BioAnalyzer. Libraries were sequenced at the NUSeq Core Facility of Northwestern University on an Illumina NextSeq500 instrument with single-end and 75-bp read length settings. Each group was sequenced in triplicate. For quality control, raw fastq files were pre-processed using TrimGalore (version 0.4.4) and cutadapt (version 1.14) with single-end trimming mode, Phred score cutoff of 20 and minimum sequence length cutoff of 20 bp (3) which trimmed off low-quality base calls. This was followed by adapter removal from the 3' end of the reads, resulting in filtered reads with average read depth of 24.1 million reads. Trimmed reads were aligned to the ENSEMBL human genome version GRCh38 using STAR (2.5.2) (4) and SAMtools (5). Expression quantification and count matrix generation were performed using rsem. Pre-filtering was done to keep only those genes with row totals (counts across all samples) > 10. Additional filtering was conducted to remove genes for which 70% of samples had no reads. Differential expression was performed using DESeq2, which utilizes a negative binomial distribution to model RNA-seq counts. Statistical differences were determined using Wald test. The p-values were adjusted using the Benjamini-Hochberg procedure. The differentially expressed genes included had an FDR-adjusted p-value < 0.10. Telescope was used to identify transposable elements (TE) across the genome (6). A count matrix was produced for each sample and differential expression of TEs was conducted between the pre- and post-treatment groups using DESeq2.

References

1. Teschendorff AE, Lee SH, Jones A, et al. HOTAIR and its surrogate DNA methylation signature indicate carboplatin resistance in ovarian cancer. Genome Med 2015;7:108.

2. Zhang Y, Parmigiani G, Johnson WE. ComBat-seq: batch effect adjustment for RNA-seq count data. NAR Genom Bioinform 2020;2:lqaa078.
3. Martin M. Cutadapt removes adapter sequences from high-throughput sequencing reads. EMBnetjournal 2011;17:3.
4. Dobin A, Davis CA, Schlesinger F, et al. STAR: ultrafast universal RNA-seq aligner. Bioinformatics 2013;29:7.
5. Li H, Handsaker B, Wysoker A, et al. The Sequence Alignment/Map format and SAMtools. Bioinformatics 2009;25:2078-9.
6. Bendall ML, de Mulder M, Iñiguez LP, Lecanda-Sánchez A, Pérez-Losada M, Ostrowski MA, et al. (2019) Telescope: Characterization of the retrotranscriptome by accurate estimation of transposable element expression. PLoS Comput Biol 15(9): e1006453.

Supplementary Tables:

Supplementary Table S1 – Patient characteristics

	Overall (n = 45)
Age at Registration in Years	
Median (Range)	61 (34 – 88)
Race	
Asian	2 (4.4)
Black	5 (11.)
Not Reported/Refused	1 (2.2)
Unknown	1 (2.2)
White	36 (80.0)
Ethnicity	
Hispanic or Latino	1 (2.2)
Non-Hispanic	43 (95.6)
Not Reported	2 (2.2)
Platinum resistant	45 (100)
Prior treatment regimens	
Median (Range)	5 (1 – 11)
Prior platinum-based regimens	
Median (Range)	2 (1 – 4)
Data are n (%) unless otherwise noted	

Supplementary Table S2 – Summary of adverse events and attributed drug.

			n=43
	Guadecitabine	Pembrolizumab	Either Drug
Adverse Events	40 (93.0)	32 (74.4)	41 (95.3)
Adverse Events (Grade 1-2)	39 (90.7)	31 (72.1)	40 (93.0)
Adverse Events (Grade 3-4)	21 (48.8)	8 (18.6)	24 (55.8)
Serious Adverse Events	3 (7.0)	6 (14.0)	7 (16.3)
Serious Adverse Events (Grade 1-2)	1 (2.3)	1 (2.3)	2 (4.7)
Serious Adverse Events (Grade 3-4)	3 (7.0)	5 (11.6)	6 (14.0)
			Data are n (%)

Supplementary Table S3A. Adverse events and serious adverse events related to guadecitabine.

**Adverse Event***	**Grade 1-2**	**Grade 3-4**	**Total****
Lymphocytopenia	20 (46.5%)	6 (14.0%)	26 (60.5%)
Leukopenia	13 (30.2%)	12 (27.9%)	25 (58.1%)
Neutropenia	5 (11.6%)	16 (37.2%)	21 (48.8%)
Anemia	19 (44.2%)		19 (44.2%)
Fatigue	14 (32.6%)	1 (2.3%)	15 (34.9%)
Injection site reaction	12 (27.9%)		12 (27.9%)
Investigations - Other	9 (20.9%)		9 (20.9%)
Nausea	6 (14.0%)		6 (14.0%)
Anorexia	5 (11.6%)		5 (11.6%)
Arthralgia	5 (11.6%)		5 (11.6%)
Thrombocytopenia	5 (11.6%)		5 (11.6%)
Diarrhea	3 (7.0%)		3 (7.0%)
**Serious Adverse Event**	**Grade 1-2**	**Grade 3-4**	**Total****
Febrile neutropenia		1 (2.3%)	1 (2.3%)
Neutropenia		1 (2.3%)	1 (2.3%)
Otitis media		1 (2.3%)	1 (2.3%)
Sinusitis	1 (2.3%)		1 (2.3%)
Skin infection		1 (2.3%)	1 (2.3%)
*Adverse events occurring in >5% of patients listed<br>**No grade 5 events			

Supplementary Table S3B. Adverse events and serious adverse events related to pembrolizumab.

Adverse Event*	Grade 1-2	Grade 3-4	Total**
Fatigue	10 (23.3%)	1 (2.3%)	11 (25.6%)
Transaminitis (ALT)	7 (16.3%)		7 (16.3%)
Diarrhea	7 (16.3%)		7 (16.3%)
Dry skin	6 (14.0%)		6 (14.0%)
Nausea	6 (14.0%)		6 (14.0%)
Arthralgia	5 (11.6%)		5 (11.6%)
Pruritis	5 (11.6%)		5 (11.6%)
Abdominal pain	4 (9.3%)		4 (9.3%)
Transaminitis (AST)	4 (9.3%)		4 (9.3%)
Colitis	3 (7.0%)	1 (2.3%)	4 (9.3%)
Lymphocytopenia	4 (9.3%)		4 (9.3%)
Maculo-papular rash	3 (7.0%)	1 (2.3%)	4 (9.3%)
Leukopenia	2 (4.7%)	2 (4.7%)	4 (9.3%)
Anorexia	3 (7.0%)		3 (7.0%)
Chills	3 (7.0%)		3 (7.0%)
Vomiting	3 (7.0%)		3 (7.0%)
Weight loss	3 (7.0%)		3 (7.0%)
**Serious Adverse Event**	**Grade 1-2**	**Grade 3-4**	**Total****
Colitis	1 (2.3%)	1 (2.3%)	2 (4.7%)
Arthritis		1 (2.3%)	1 (2.3%)
Febrile neutropenia		1 (2.3%)	1 (2.3%)
Neutropenia		1 (2.3%)	1 (2.3%)
Thromboembolic event		1 (2.3%)	1 (2.3%)
*Adverse events occurring in >5% of patients listed<br>**No grade 5 events			

Supplementary Table S4. Top 50 differentially upregulated genes after guadecitabine + pembrolizumab treatment of ovarian cancer patients (n=9 paired samples). mRNA levels were measured by RNAseq.

Gene Name	log2 Fold Change	FDR-adjusted p-value
IFNG	3.021943597	0.000807
PNMA5	2.911491263	0.013305
CXCL9	2.611062621	2.05E-07
ACOD1	2.574988025	0.084935
IL21	2.443231297	0.017989
CT45A5	2.436280294	0.023332
IDO1	2.423100365	2.54E-06
FPR2	2.422398678	0.045380
PLA2G2D	2.412921216	2.18E-05
GZMH	2.268437744	2.79E-06
GZMK	2.244753795	0.001769
GZMA	2.160537063	9.83E-05
CD8A	2.157331338	7.06E-06
CALHM6	2.124869018	8.27E-07
CXCR6	2.118103195	2.18E-05
HTRA4	2.094718796	0.000315
PDCD1	2.076223387	1.27E-06
CASP5	2.061952936	0.025993
SIGLEC8	2.046599027	0.002471
IGF2BP1	1.972021776	0.013133
CD8B	1.966866185	0.018818
LAG3	1.961054573	0.002136
GPR171	1.950795401	0.000797
CCL18	1.940577946	1.87E-05
GBP5	1.933054707	5.67E-07
ADAMDEC1	1.916304502	2.83E-07
TLR8	1.884197324	0.001496
TMIGD3	1.869853443	0.004575
PRR5-ARHGAP8	1.828943358	0.089720
PRF1	1.827549639	0.000189
KCNJ10	1.818239279	7.63E-05
GZMB	1.800743139	0.070990
SLAMF7	1.795691466	0.000108
CD2	1.773541842	2.41E-05
NKG7	1.758246502	0.005008
CHIT1	1.755242023	0.004425
CXCL11	1.739105067	0.001274
FCGR1CP	1.73851812	0.000838
C1QB	1.733936177	1.13E-09
GPR84	1.728380317	0.001041
CLEC4E	1.719569177	6.54E-05
SIGLEC10	1.715704444	8.27E-07
PDCD1LG2	1.713103785	0.000145
FCGR1A	1.708495598	6.78E-06
TIFAB	1.702393538	0.025508
TIGIT	1.696611575	0.000677
LINC02446	1.694817585	0.050489
PLA2G7	1.682182086	0.002040
CCL5	1.68198303	0.001796
DLGAP1	1.671422442	0.045647

Supplementary Table S5. Differentially downregulated genes (41 total) after guadecitabine + pembrolizumab treatment of ovarian cancer patients (n=9 paired samples). mRNA levels were measured by RNAseq.

Gene Name	log2 Fold Change	FDR-adjusted p-value
PGAP2	-0.345407592	0.035958496
NKIRAS1	-0.367687668	0.081703089
SRSF5	-0.380340907	0.028366191
ZNF655	-0.398602078	0.003168626
SDHAP1	-0.410338197	0.074571972
HSD17B8	-0.421765695	0.098659091
CREBZF	-0.45580986	0.077839995
NREP	-0.456134357	0.000669948
L3HYPDH	-0.458217421	0.017157476
SGSM2	-0.506855608	0.034421708
SNHG14	-0.528938406	0.03754422
RNF208	-0.57986596	0.080460579
ZNF321P	-0.601373661	0.010497706
NSUN5P1	-0.662756505	0.094221581
TIMP3	-0.676978243	0.083678637
MIRLET7A1HG	-0.746668684	0.095702681
DNM1	-0.748204932	0.016338599
VAMP1	-0.759141863	0.007033378
CPLX1	-0.866386115	0.016158171
RPL10P9	-1.07217148	0.08247558
GABRD	-1.141079875	0.052367756
CARMN	-1.153806166	0.081926728
C1QTNF3	-1.161311733	0.07190082
AC135050.2	-1.168996494	0.067990322
NALT1	-1.296956295	0.048926277
LINC01238	-1.363348944	0.07190082
AC131649.2	-1.474638517	0.035958496
FLG	-1.485695252	0.032600299
AC092535.5	-1.643213806	0.050448743
FGF14	-1.748033355	0.084152885
KCNJ13	-1.776394198	0.014155277
AC027601.3	-1.797433312	0.060255252
COX6A2	-2.020299371	0.07190082
HMCN2	-2.194654715	0.042789583
SNORD3D	-2.430748836	0.089551416
RELN	-2.591988519	0.070144361
ACADL	-2.748984955	0.002128598
AC087164.2	-2.944168458	0.011618544
MYH1	-3.092287617	0.018818117
AC090517.4	-3.424373847	0.041357655
FGB	-3.533332606	0.001274024

Supplementary Table S6. Top 50 differentially upregulated genes measured before guadecitabine + pembrolizumab treatment in durable CBR patients (n=9) compared with non-responders (n=7). mRNA levels were measured by RNAseq.

Gene Name	log2 Fold Change	FDR-adjusted p-value
ALPG	8.85098489	0.000691701
COX8C	8.566262493	0.042625388
ALPP	8.278134784	0.00252245
GPR1-AS	7.533349103	0.009387357
PRSS41	7.409782478	0.001119237
IGKV1D-17	5.969474335	0.022648315
PKHD1L1	5.832515885	0.000260587
RAG2	5.826777943	0.022786167
LTF	5.769440902	0.000401743
CCL21	5.452005367	0.012875747
IGKV6-21	5.399432873	0.034681608
MS4A8	5.355262404	0.052538535
ZNF716	5.311466305	0.058541591
CLEC2L	5.181111018	0.018998875
NR5A1	4.966615821	0.055338403
OR56A3	4.885062642	0.054268007
RPL23AP60	4.815864593	0.05917125
PGC	4.700403267	0.049572273
CEACAM6	4.603132218	0.009387357
CD1B	4.478789883	0.098557991
AKR1B15	4.456120318	0.037946087
AC013457.1	4.436407013	0.037946087
AC096656.1	4.389959555	0.059428475
PTCRA	4.315932565	0.021976816
AC073612.1	4.261104874	0.034681608
RRN3P4	4.184449796	0.052538535
LINC02257	4.171797184	0.00667502
TACSTD2	4.15290248	0.000260587
AL022342.1	4.150202265	0.052538535
C8orf34-AS1	3.962821145	0.057504971
LINC01133	3.886653558	0.083309771
DDX43	3.879218215	0.046217468
TRDC	3.867880253	0.031582847
MUC5B	3.840106316	0.015982214
AC091544.2	3.77761556	0.087473287
AC019131.3	3.74758465	0.063639948
TEX41	3.676142164	0.063110924
SLC25A48	3.658013778	0.025467258
UPK3B	3.648086999	0.023356137
ELAPOR1	3.553423218	0.044374432
CSF2	3.530921675	0.048742093
KRT15	3.490924213	0.064784806
PCDHGB5	3.475700344	0.00127941
HBB	3.445634317	0.033830493
GCNT1P3	3.430001058	0.083545384
LINC01226	3.422868569	0.079177409
RAG1	3.417070941	0.001119237
STOML3	3.412825384	0.091567883
EYA2	3.408922836	0.015982214

Supplementary Table S7. Top 50 differentially dowregulated genes measured before guadecitabine + pembrolizumab treatment in durable CBR patients (n=9) compared with non-responders (n=7). mRNA levels were measured by RNAseq.

Gene Name	log2 Fold Change	FDR-adjusted p-value
ZG16	-9.159804931	0.055338403
NXF2	-8.420283058	0.002484651
TTR	-8.326617288	0.054268007
CYP2B6	-8.190694444	0.042634455
ALB	-8.110597445	0.016759077
ADH1A	-8.088131576	0.006332901
PIK3C2G	-7.776423252	0.073433344
BAAT	-7.643517215	0.016759077
SERPIND1	-7.337692859	0.016759077
CYP4F2	-7.266118759	0.092045328
ADH1C	-7.249112267	0.015982214
AHSG	-7.211869541	0.095277598
UGT2B4	-7.203896338	0.09862059
HRG	-7.130767014	0.026889926
UROC1	-7.116128569	0.016759077
AKR1C4	-6.894481198	0.031582847
AC061961.1	-6.425141361	0.061646249
HNF4A	-6.272416001	0.068086988
APOC4-APOC2	-6.270267031	0.065612319
CLRN1-AS1	-6.265758468	0.061991483
PON1	-6.26474166	0.016759077
CLRN3	-6.221788263	0.02729617
CA5A	-6.188333223	0.049006108
UPB1	-6.184798914	0.083169573
C2CD4A	-6.16705116	0.002189684
COL2A1	-6.141009492	0.006541502
PLPPR1	-6.068353441	0.019060092
ADH1B	-6.028003518	0.029903832
AL583836.1	-6.018234934	0.071747497
CES5A	-5.945791283	0.06606444
ABCB11	-5.86816446	0.05536931
GSTA1	-5.821804649	0.015982214
UGT2A1	-5.802276023	0.058048503
HABP2	-5.776185063	0.058048503
ETNPPL	-5.774276708	0.019396141
RIPPLY1	-5.773658431	0.052687211
NR0B2	-5.616172643	0.033830493
IYD	-5.604883809	0.031582847
RHBG	-5.517500627	0.016759077
NAT8	-5.498378041	0.033830493
LINC01564	-5.492564017	0.035708906
FTCD	-5.461714653	0.044249469
SLC38A3	-5.429165211	0.016759077
TM4SF4	-5.332323078	0.070812194
GPX2	-5.240872035	0.034681608
TREH	-5.219195809	0.068122133
AC063919.1	-5.200234276	0.052766685
NPC1L1	-5.195610435	0.021976816
LINC01554	-5.166978616	0.022786167

Supplementary Table S8: List of transposable elements (TE) differentially expressed (upregulated and downregulated) between pre- and post-treatment tumor specimens (FDR<0.05).

TE	FDR pvalue	Fold-Change (post vs. pre)
HERVW_11p14.2	0.077155907	8.788342658
HML6_12p11.21	0.084432576	8.416017928
HERVH_Xq23b	0.056078697	8.251313234
HERVL40_4q22.2	0.065079534	7.408100996
HERVH_11q22.2	0.021983366	6.009709729
HERVFH19_12p11.21	0.084040231	5.221384318
ERV316A3_14q21.1c	0.035369708	4.915629252
MER34B_11p15.5	0.043686343	4.51033525
HERVE_8p23.1e	0.063799393	4.281101183
HML5_18q21.2	0.05227583	3.602453103
HERVL_10p15.1	6.29E-09	0.018576868
HERVH_17q24.2	3.88E-05	0.044694473
MER4_14q11.2a	0.080517989	0.064412481
HML3_4q22.1a	0.098489402	0.126179919
HUERSP2_3q26.33	0.09896083	0.13634798
HML5_12q15	0.095837113	0.136470291
HERVH_21q21.1b	0.065524387	0.160529814
HERVH_3q11.1	0.061353986	0.17240441
ERVLE_15q21.2	0.060836837	0.177612811
MER4_3q29e	0.062841848	0.179679473
HERVK11_22q11.21	0.070394985	0.279846533

Supplementary Table S9. Antibodies used for CyTOF analysis.

Target	Metal-conjugate	Clone
Live and Dead	In115	
CD57	In113	HCD57
CD19	Nd142	SJ25C1
CD4	Nd143	SK3
CD8	Nd144	SK1
IgD	Nd146	IA6-2
CD85j	Sm147	292319
CD11c	Nd148	Bu15
CD16	Sm149	3G8
CD3	Nd150	UCHT1
CD38	Eu151	HB-7
CD27	Sm152	L128
CD11b	Eu153	ICRF44
CD14	Sm154	M5E2
CCR6	Gd155	G034E3
CD94	Gd156	HP-3D9
CD86	Gd157	IT2.2
CXCR5	Gd158	RF8B2
CXCR3	Tb159	G025H7
CCR7	Gd160	150503
CD45RA	Dy162	HI100
CD20	Dy164	2H7
CD127	Ho165	A019D5
CD33	Er166	P67.8
CD28	Er167	L293
CD24	Er168	ML5
ICOS	Tm169	DX29
CD161	Er170	DX12
TCRgd	Yb171	B1
PD-1	Yb172	EH12.1
CD123	Yb173	9F5
CD56	Yb174	NCAM16.2
HLA-DR	Lu175	G46-6
CD25	Yb176	M-A251

Supplementary Table S10. Antibodies used for CyTEK analysis.

Target	Conjugate	Clone	Cat#	Vendor
Live and Dead	Zombie yellow		423104	BioLegend
CD14	Alexa Fluor 647	MφP9	562690	BD Biosciences
CD8a	Alexa Fluor 700	RPA-T8	301028	BioLegend
CD3	Spark Blue™ 550	SK7	344852	BioLegend
CD19	APC	HIB19	302212	BioLegend
CD38	PerCP	HIT2	303520	BioLegend
CD4	PE	RPA-T4	300539	BioLegend
CD11c	BV480	B-ly6	566184	BD Biosciences
CD16	BV785	3G8	302046	BioLegend
CD127 (IL-7Ra)	PE/CY7	A019D5	351320	BioLegend
TCRgd	PerCP5.5	B1	331224	BioLegend
CD27	APC/Fire™810	L128	393214	BioLegend
CD274 (PD-L1)	BV711	29E.2A3	329722	BioLegend
CD28	BV650	CD28.2	302946	BioLegend
CD33	BV570	WM53	303417	BioLegend
CD197 (CCR7)	BV421	G043H7	353208	BioLegend
CD45RA	BV510	HI100	304142	BioLegend
CD25 (IL-2R)	PE/Dazzle 594	M-A251	356126	BioLegend
HLA-DR	BV750	L243	307672	BioLegend
CD279 (PD-1)	Alexa Fluor 488	EH12.2H7	329936	BioLegend
CD56 (NCAM)	APC/CY7	5.1H11	362512	BioLegend
CD11b (Mac-1)	Pacific Blue	M1/70	101224	BioLegend
CD45	Spark NIR™ 685	2D1	368552	BioLegend

Supplementary Table S11. Antibodies used for mIHC.

	Antigen	Primary antibodies			Fluorophore
Panel 1		Dilution	Vendor	Catalog #	
	CD3	1:1	Biocare medical	PP215AA	Opal 520
	CD8	1:200	Cell Signaling	70306S	Opal 540
	CD20	1:1	Biocare medical	PM004AA	Opal 570
	CD68	1:1	Biocare medical	PM033AA	Opal 620
	Foxp3	1:100	BioLegend	320102	Opal 650
	PanCK	1:200	Abcam	Ab27988	Opal 690
Panel 2					
	A2AR	1:200	Sigma	HPA075997	Opal 520
	CD8	1:200	Cell Signaling	70306S	Opal 620
	PD-L1	1:200	Cell Signaling	13684S	Opal 570
	NY-ESO-1	1:200	Invitrogen	35-6200	Opal 650
	PanCK	1:200	Abcam	Ab27988	Opal 690

Supplementary Figures:

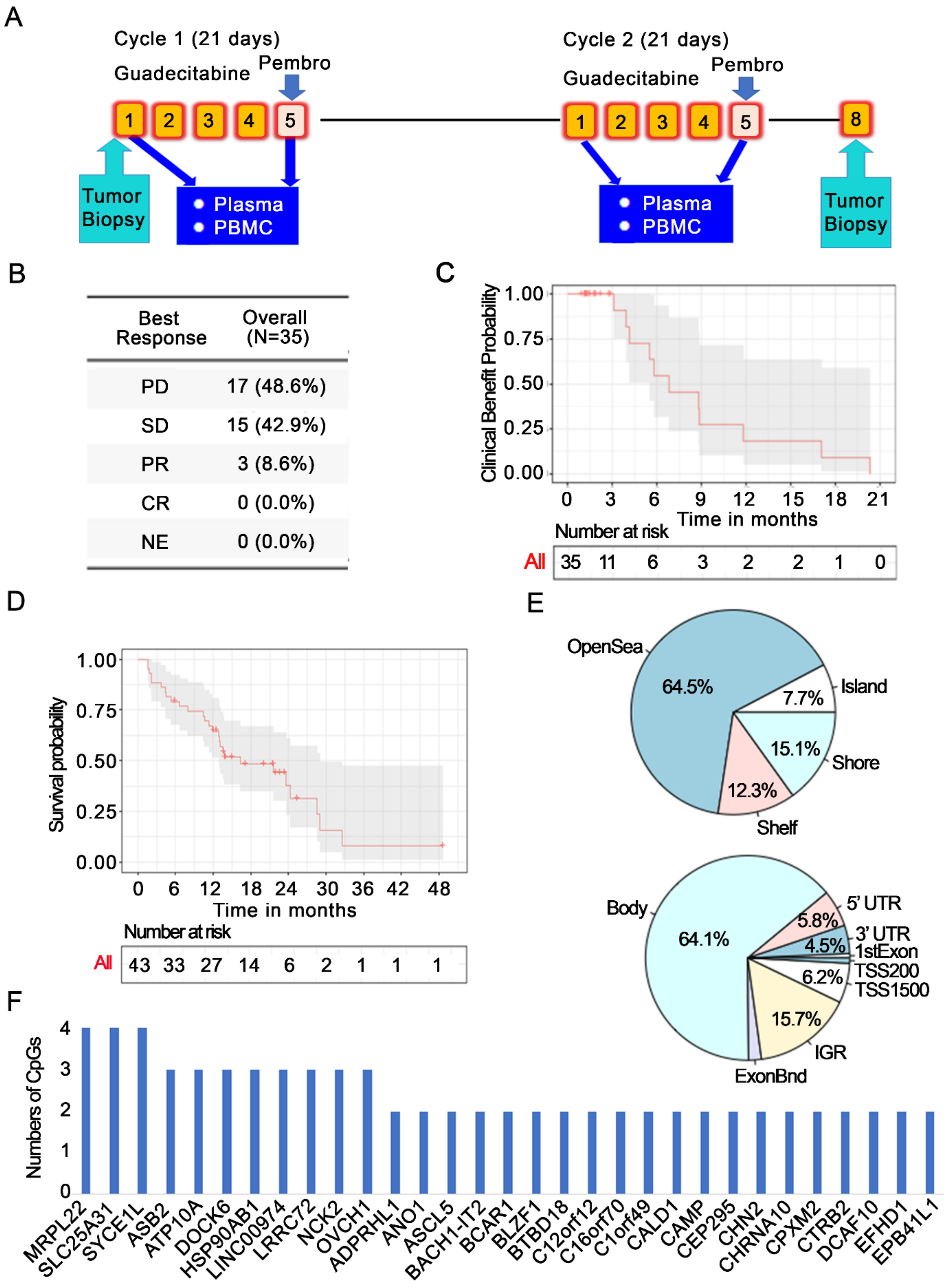

Supplementary Figure S1. Results of clinical trial: A, Treatment schema. **B**. Summary of clinical responses. **C.** Duration of clinical benefit (months). **D.** Kaplan Meier curve illustrates overall survival (n=43). **E**, Distribution of differentially methylated CpGs in patient samples after guadecitabine+prembrolizumab treatment (C2D8) vs. before treatment (C1D1). **F**, Genes with highest number of differentially methylated CpGs in the promoter-associated region (TSS200+TSS1500) in C2D8 compared with C1D1 tumor biopsies (n=11 pairs). Pembro, pembrolizumab.

A

PATHWAY	FDR q-value
GLUCURONIDATION	0.027482174
MRNA_PROCESSING	0.01865597
STRIATED_MUSCLE_CONTRACTION_PATHWAY	0.013170967
TCA_CYCLE_AKA_KREBS_OR_CITRIC_ACID_CYCLE	0.022824746
METABOLIC_REPROGRAMMING_IN_COLON_CANCER	0.022776695
ELECTRON_TRANSPORT_CHAIN_OXPHOS_SYSTEM_IN_MITOCHONDRIA	0.028358208
OXIDATIVE_PHOSPHORYLATION	0.032652568
PPAR_SIGNALING_PATHWAY	0.029365905
MITOCHONDRIAL_COMPLEX_1_ASSEMBLY_MODEL_OXPHOX_SYSTEM	0.033290487

B

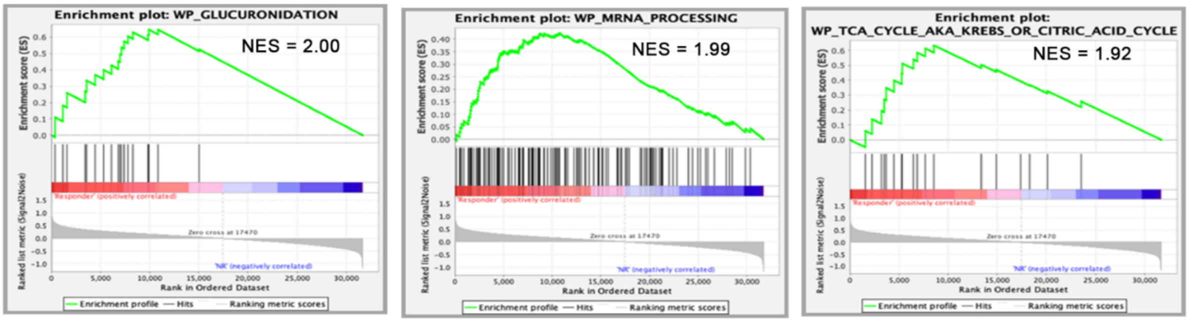

Supplementary Figure S2. Differences in gene expression between durable CBR and non-responders. A. Top ten enriched biological pathways by statistical significance (FDR q-value) identified by GSEA of gene expression in C1D1 tumors from durable CBR vs. non-responder patients. **B.** GSEA enrichment plots for the pathways “Glucuronidation”, “mRNA processing”, and “TCA cycle aka Krebs or citric acid cycle”.

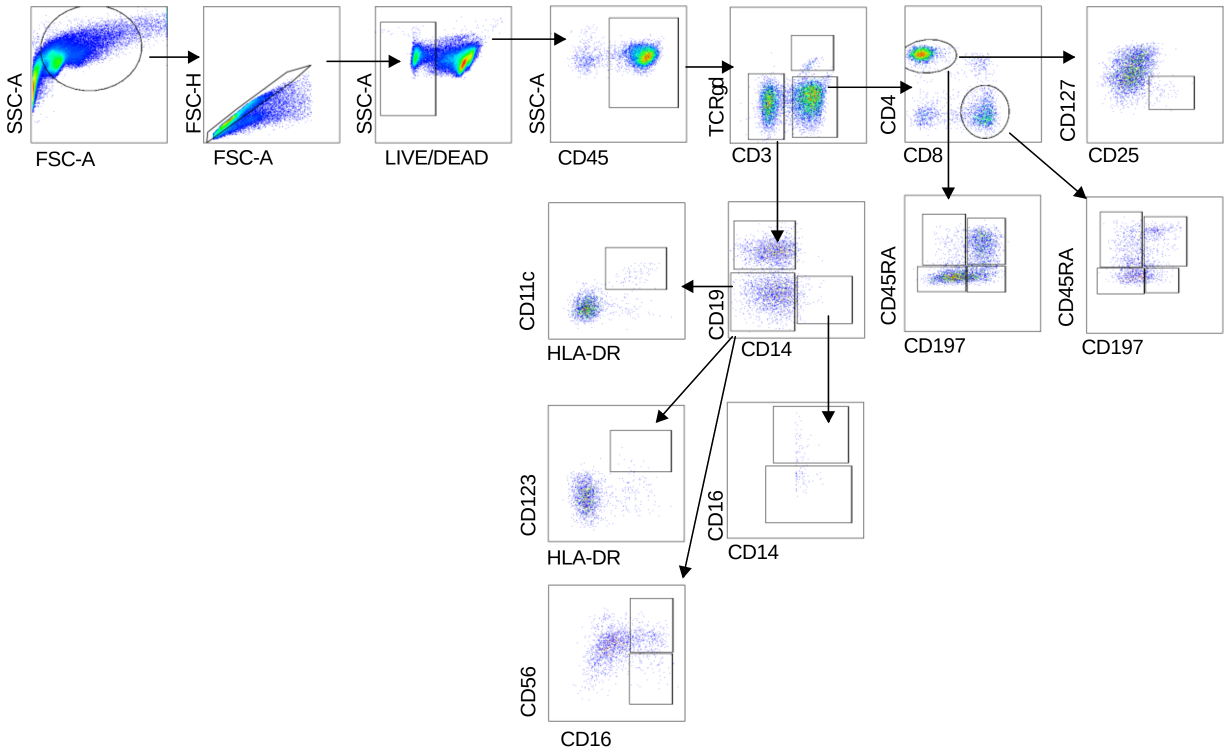

Supplementary Figure S3. Gating strategies for the main immune populations in PBMC. Single cell suspensions of PBMC were stained and analyzed by CyTEK for measuring CD4+ T cells (CD3+CD4+), CD8+T cells (CD3+CD8+), B cells (CD19+CD20+), classic monocytes (Lin-CD14+CD16-), non-classical (NC) monocytes (Lin-CD14^{low}CD16+), monocytic dendritic cells (mDC), plasmacytoid dendritic cells (pDC), CD56^{hi} and CD56^{dim} NK cells.

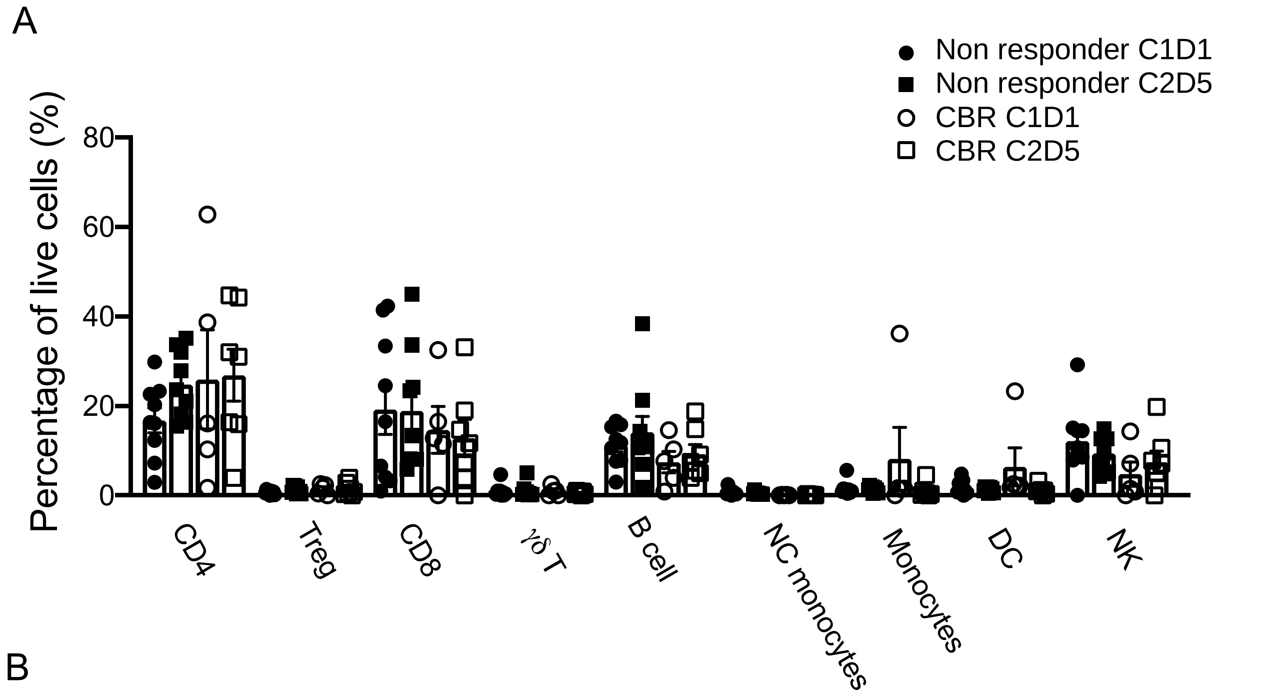

Supplementary Figure S4. The immune profiling of PBMCs from C1D1 and C2D5. A. Frequencies of different immune populations in the PBMC from the extended cohort of non-responders (n=9) and durable CBR (n=6) at C1D1 and C2D5 analyzed by CyTEK. **B**. Frequencies of different T cell subsets indicated from PBMC of non-responders (n=9) and durable CBR (n=6) at C1D1 and C2D5 analyzed by CyTEK.

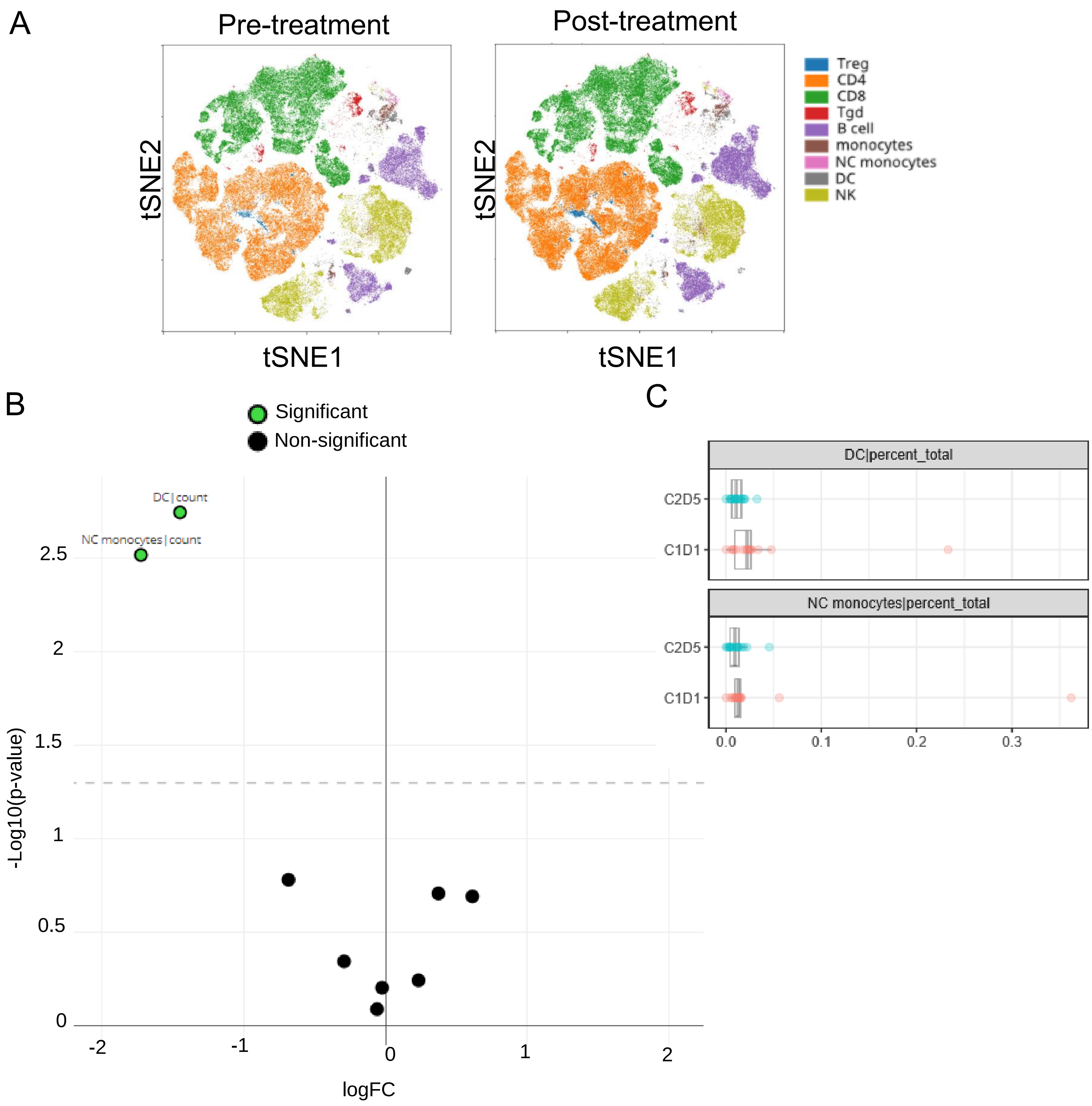

Supplementary Figure S5. Identification of populations with significant differences between pre- and post-treatment with guadecitabine + pembrolizumab. A, Exemplified tSNE visualization of overlaid cell population composition in the PBMCs of durable CBR (n=6) between C1D1 and C2D5. **B.** EdgeR analysis identified PBMC populations in durable CBR (n=6) with significant differences in relative abundance between C1D1 and C2D5. EdgeR

analysis identified population that show significant difference between C1D1 and C2D5. **C**. Abundance plots for significant clusters of DC and NC monocytes from PBMCs of durable CBR (n=6) between C1D1 and C2D5 by SAM CITRUS analysis.

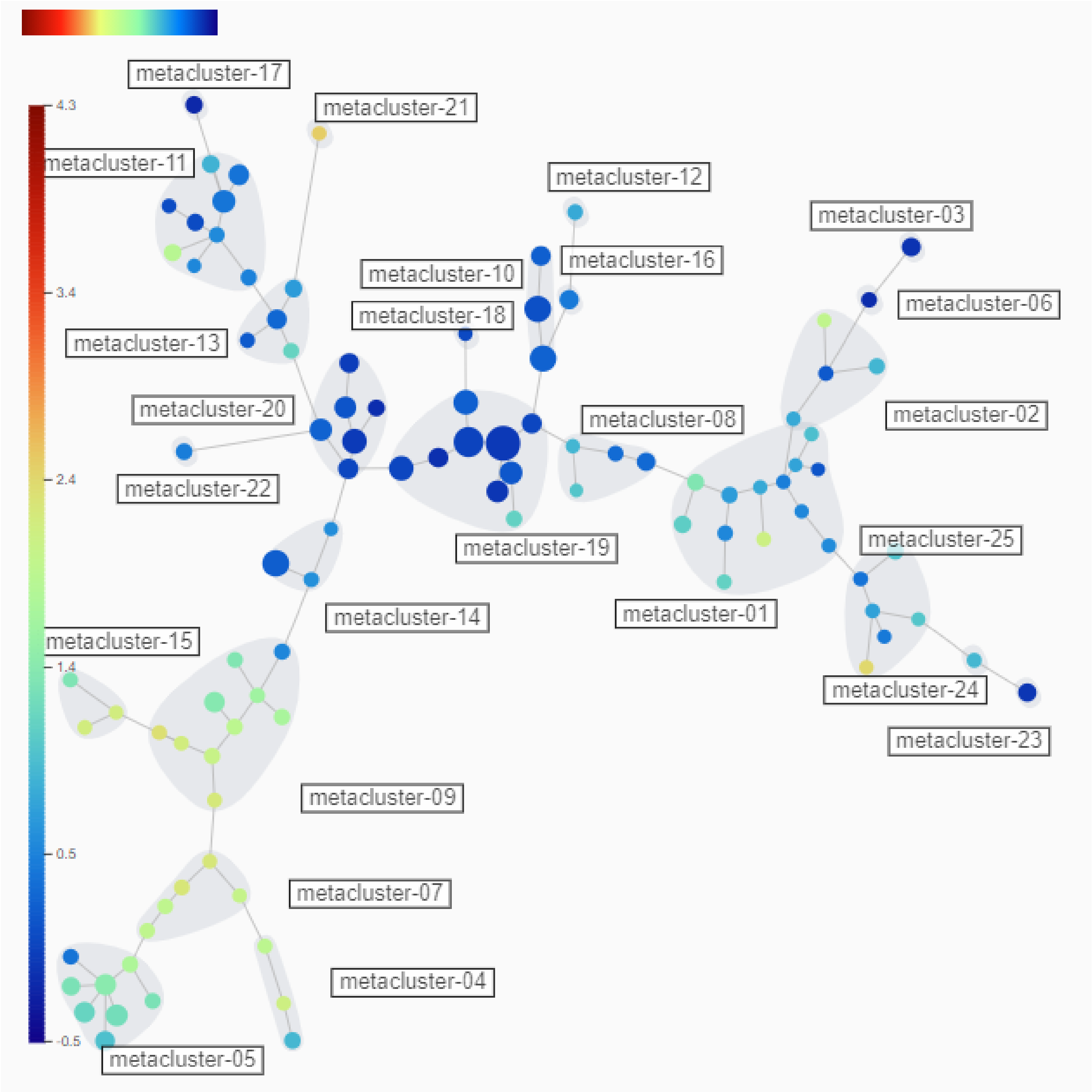

Supplementary Figure S6. The FlowSOM tree for the PBMC of patients at C1D1 showing the unsupervised metaclustering. The background coloring indicates the relative abundance of each metacluster.

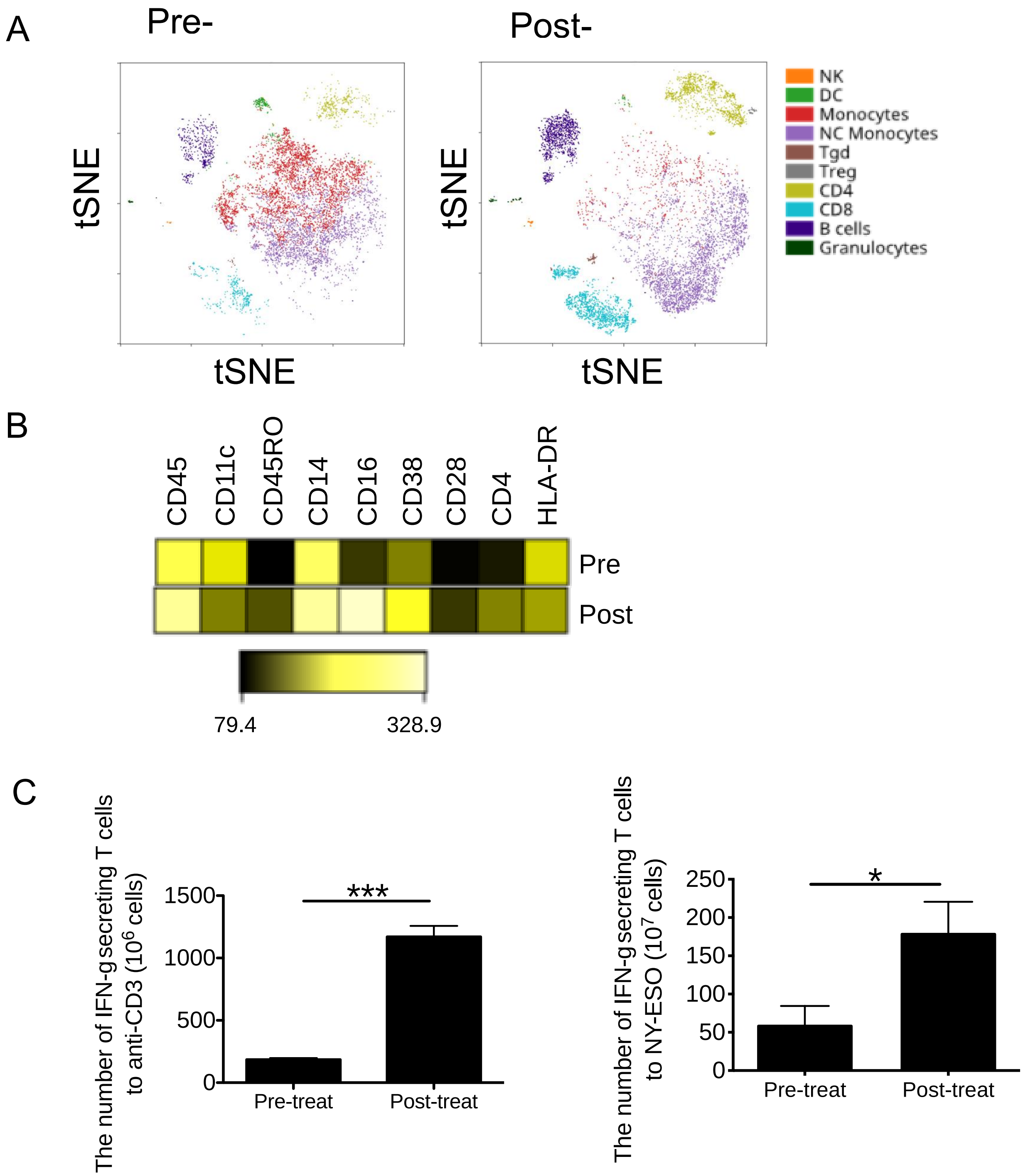

Supplementary Figure S7. Identification of differences in ascites from patient with durable CBR before and after treatment with guadecitabine + pembrolizumab. A. Exemplified tSNE visualization of overlaid immune cell population composition in the ascites from a durable CBR before and after treatment. **B.** The heat map represents the median expression levels of indicated markers within $CD45^+$ cells from the ascites. **C.** ELISPOT analysis of IFN-γ secreting T cells from tumor cell depleted ascites treated as indicated in the presence of anti-CD3 or NY-ESO-1

peptides. The total number of IFN-γ secreting T cells was counted. Error bars represent mean with SD. *p < 0.05, ***p < 0.001. The p-values were calculated using two-sided t-tests.

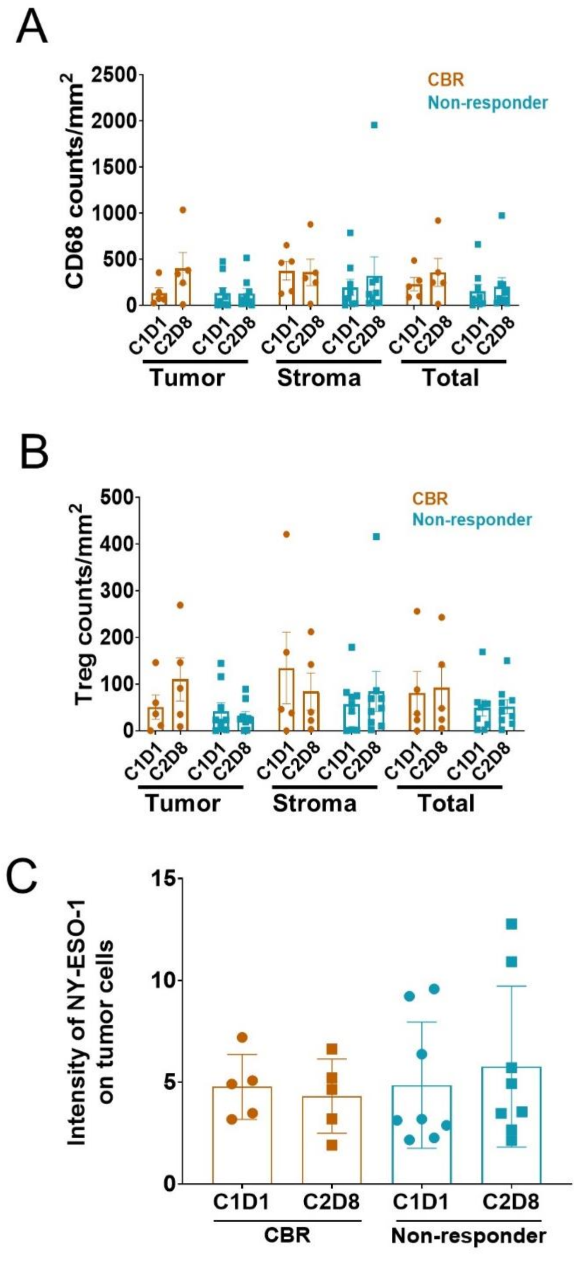

Supplementary Figure S8. A, B. Density of tumor infiltrating CD68+ macrophages (A) and Treg cells (B) in the compartments of tumor-nest and stroma from durable CBR (n=5) and non-responders (n=9) determined by mIHC analysis. **C.** Expression levels of NY-ESO-1 on PanCK^{+} tumor cells at C1D1 and C2D8 between durable CBR (n=5) and non-responders (n=9).

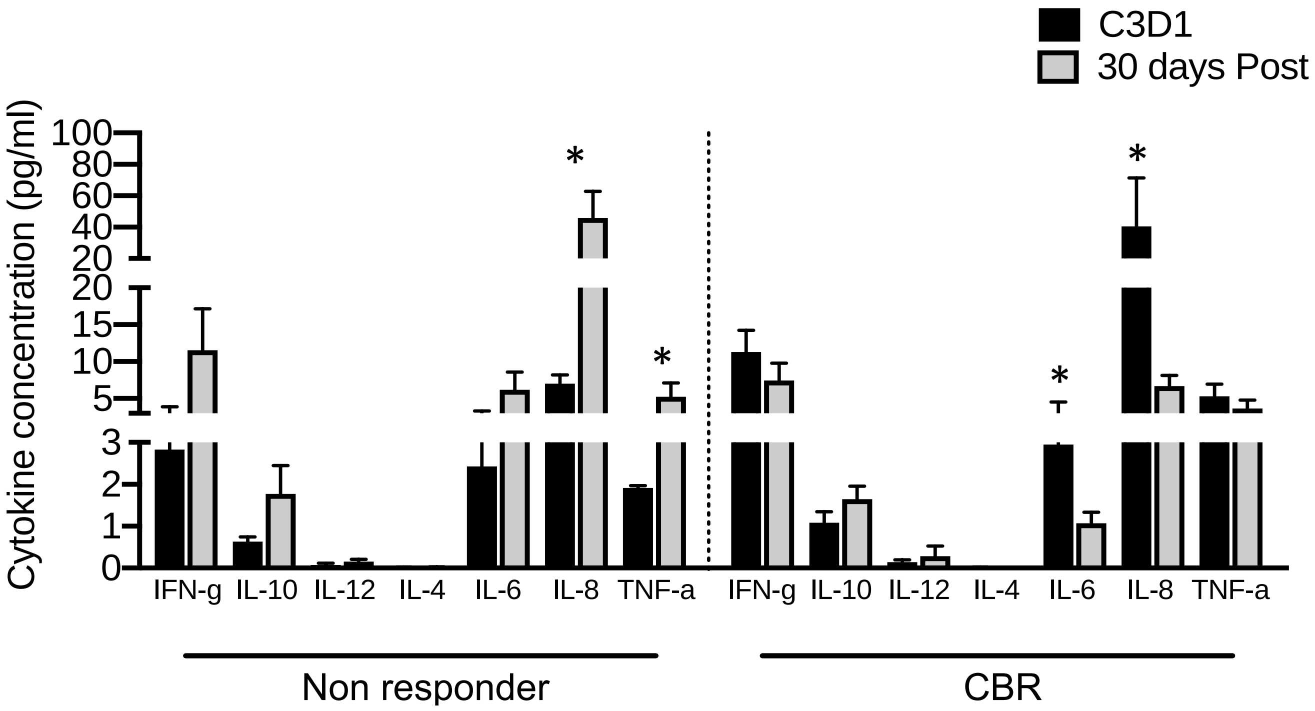

Supplementary Figure S9. Plasma levels of proinflammatory cytokines between non-responders and durable CBR after treatment with guadecitabine + pembrolizumab. The levels of plasma cytokines as indicated were measured by ELISA from non-responders (n=12) and durable CBR (n=5). Values are presented as means ± SD. * $p < 0.05$, ** $p < 0.01$. The p-values were calculated using two-sided t-tests.
